# Interventions to increase personal protective behaviours to limit the spread of respiratory viruses: A rapid evidence review and meta‐analysis

**DOI:** 10.1111/bjhp.12542

**Published:** 2021-06-26

**Authors:** Olga Perski, Dorothy Szinay, Elizabeth Corker, Lion Shahab, Robert West, Susan Michie

**Affiliations:** ^1^ Department of Behavioural Science and Health University College London UK; ^2^ Behavioural and Implementation Science Group School of Health Sciences University of East Anglia Norwich UK; ^3^ Department of Clinical, Educational and Health Psychology University College London UK

**Keywords:** behaviour change, COVID‐19, intervention, personal protective behaviours, rapid review, SARS‐CoV‐2

## Abstract

**Purpose:**

Increasing personal protective behaviours is critical for stopping the spread of respiratory viruses, including SARS‐CoV‐2: We need evidence to inform how to achieve this. We aimed to synthesize evidence on interventions to increase six personal protective behaviours (e.g., hand hygiene, face mask use, maintaining physical distancing) to limit the spread of respiratory viruses.

**Methods:**

We used best practice for rapid evidence reviews. We searched Ovid MEDLINE and Scopus. Studies conducted in adults or children with active or passive comparators were included. We extracted data on study design, intervention content, mode of delivery, population, setting, mechanism(s) of action, acceptability, practicability, effectiveness, affordability, spill‐over effects, and equity impact. Study quality was assessed with Cochrane’s risk‐of‐bias tool. A narrative synthesis and random‐effects meta‐analyses were conducted.

**Results:**

We identified 39 studies conducted across 15 countries. Interventions targeted hand hygiene (*n* = 30) and/or face mask use (*n* = 12) and used two‐ or three‐arm study designs with passive comparators. Interventions were typically delivered face‐to‐face and included a median of three behaviour change techniques. The quality of included studies was low. Interventions to increase hand hygiene (*k* = 6) had a medium, positive effect (*d* = .62, 95% CI = 0.43–0.80, *p *< .001, *I* ^2 ^= 81.2%). Interventions targeting face mask use (*k* = 4) had mixed results, with an imprecise pooled estimate (OR = 4.14, 95% CI = 1.24–13.79, *p *< .001, *I* ^2 ^= 89.67%). Between‐study heterogeneity was high.

**Conclusions:**

We found low‐quality evidence for positive effects of interventions targeting hand hygiene, with unclear results for interventions targeting face mask use. There was a lack of evidence for most behaviours of interest within this review.

**Statement of Contribution**

***What is already known on this subject?***

- Widespread adoption of personal protective behaviours (PPBs) is needed to block respiratory viral transmission.
- Effective interventions that increase PPBs primarily target health care professionals.
- The generalizability of such interventions to the general population remains unclear.

***What does this study add?***

- This rapid review extends findings from health care to community settings.
- We found evidence for a medium, positive effect of hand hygiene interventions.
- Interventions targeting face mask use had unclear results.
- Few relevant studies were identified, and study quality was low.

## Background

Respiratory viruses such as influenza, respiratory syncytial virus, parainfluenza, rhinovirus, coronavirus (including SARS‐CoV‐2), and adenovirus enter the body through the eyes, nose, and mouth (the ‘T‐Zone’) (Killingley & Nguyen‐Van‐Tam, 2013; West, Michie, Rubin, & Amlôt, 2020). Changing human behaviour is critical for stopping the spread of respiratory viruses in general and the SARS‐CoV‐2 virus in particular, and for supporting the easing of financially and psychologically costly physical distancing measures during viral epidemics (Ferguson et al., 2006; Michie, Rubin, & Amlôt, 2020; Michie, West, & Amlôt, 2020; West et al., 2020). Personal protective behaviours, including hand washing, disinfecting fomites such as clothes or furniture, and face mask wearing, are advocated for limiting the spread of SARS‐CoV‐2 (Lunn et al., 2020; World Health Organization, 2019). Simply advising people to adopt these behaviours has been found to be insufficient, just as has explaining what to do and why these behaviours are necessary (Bish & Michie, 2010). Directly relevant evidence on interventions to promote adherence to personal protective behaviours in community‐dwelling children and adults is sparse but there is an urgent need to identify and synthesize what evidence does exist. Policymakers need evidence to inform the development of public health guidance and decide which interventions to prioritize. We adopted best practice for rapid evidence reviews to evaluate the acceptability, practicability, effectiveness, affordability, spill‐over effects (i.e., unintended consequences), and equity impact (the ‘APEASE’ criteria (Michie, Atkins, & West, 2014)) of interventions to increase personal protective behaviours to limit the spread of respiratory viruses.

**Figure 1:**
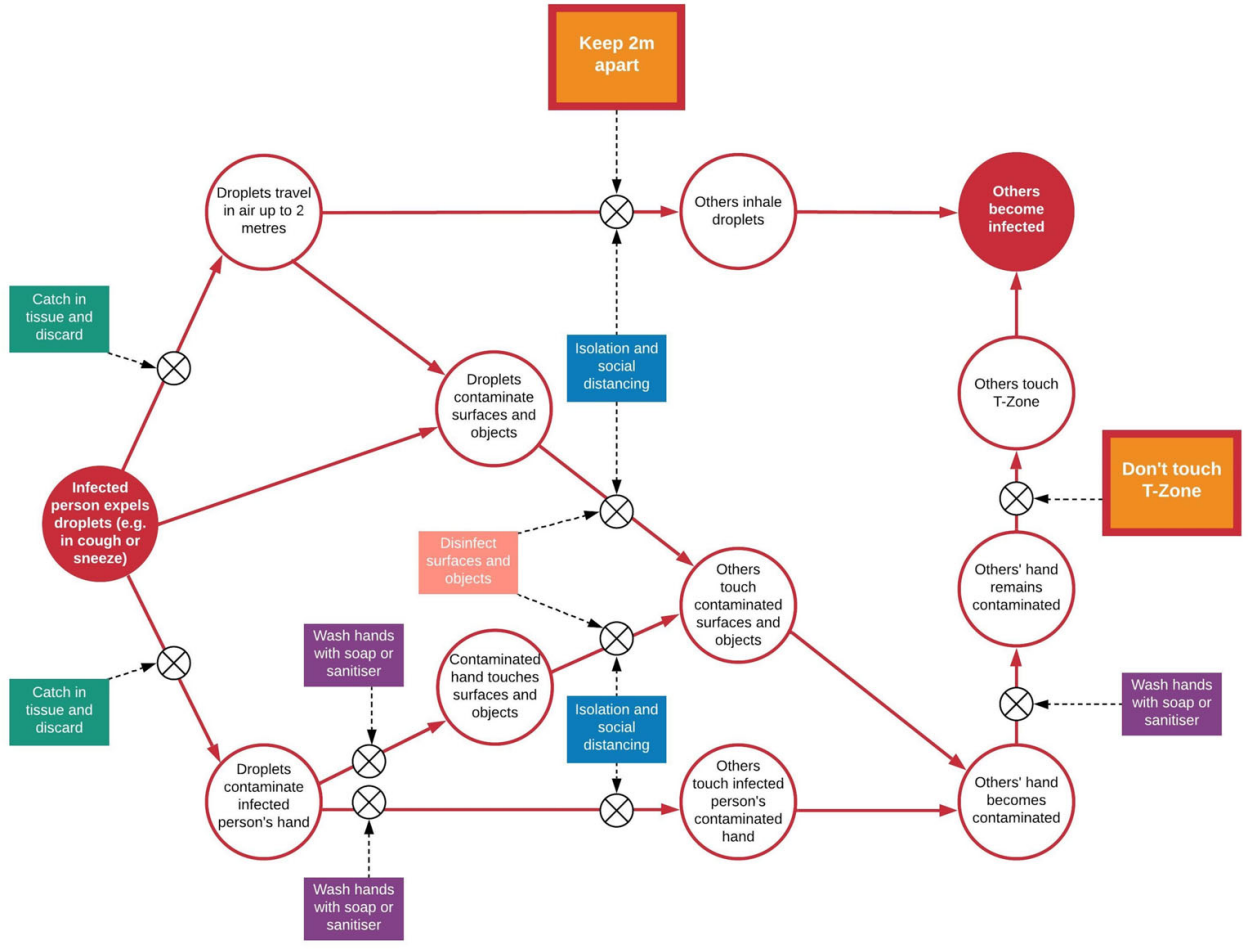
Map of personal protective behaviours relevant for blocking transmission of respiratory viruses, including SARS‐CoV‐2: hand washing and use of hand sanitizers; avoiding touching the ‘T‐Zone’; catching droplets in tissues and discarding these; face mask use; disinfecting surfaces; and maintaining physical distancing. Reproduced with permission from the authors (West & Michie, 2020).

During pandemics of respiratory viruses, multipronged approaches involving both pharmacological (e.g., vaccination) and behavioural measures (e.g., hand washing, physical distancing) are required to bring the reproductive number below 1 (Ferguson et al., 2006; Michie, Rubin, & Amlôt, 2020; Michie et al., 2020; Michie, West, Amlôt, & Rubin, 2020). Vaccination of populations will take months, even years, to roll out, especially in low‐ and middle‐income countries. Hence, physical distancing and other behavioural measures will be required, possibly permanently. Population‐wide restrictions are costly from financial, social, and psychological perspectives: The world economy has been projected to shrink by approximately 4.9% in 2020 (International Monetary Fund, 2020), with an additional 88 million people globally being pushed into extreme poverty (i.e., living on less than $1.90/day) (Blake & Wadhwa, 2020), and prolonged periods of social isolation are associated with increases in domestic violence (SafeLives, 2020) and negative mental health effects, such as post‐traumatic stress disorder, confusion, and anger (Brooks et al., 2020). Less costly, yet highly effective (Warren‐Gash, Fragaszy, & Hayward, 2012) personal protective behaviours are thus important for supporting the easing of lockdown measures to ensure long‐term suppression of viral transmission and preparedness for new viral waves and future pandemics (West et al., 2020). To successfully block the spread of respiratory viruses including (but not limited to) SARS‐CoV‐2 – which are transmitted via droplets, aerosols, and direct physical contact (Killingley & Nguyen‐Van‐Tam, 2013; West et al., 2020) – several personal protective behaviours must be adopted across the population (see Figure 1). Although systematic reviews of interventions to change hand hygiene in health care professionals are available (Edwards et al., 2012; Huis et al., 2012; Luangasanatip et al., 2015; Mbakaya, Lee, & Lee, 2017; Olena Doronina, Jones, Martello, Biron, & Lavoie‐Tremblay, 2017), generalizability to community settings is limited. There also appears to be little evidence about interventions to change behaviours such as not touching the T‐Zone (eyes, nose, and mouth), which would have a significant effect if adopted (Kwok, Gralton, & McLaws, 2015) and carry little or no costs to people or society. If adopted at scale across the population including disadvantaged communities, such interventions have the potential to reduce health inequalities. Here, we aimed to conduct a rapid evidence review to evaluate the acceptability, practicability, effectiveness, affordability, spill‐over effects, and equity of interventions to increase personal protective behaviours that limit the spread of respiratory viruses.

## Methods

### Study design

The study protocol was pre‐registered on the Open Science Framework (https://osf.io/7cphy/). During ongoing pandemics, the World Health Organization recommends the use of rapid evidence reviews for swift knowledge generation (World Health Organization, 2017). We adopted acknowledged best practice for rapid evidence reviews, which involved completing the review in a timely fashion, limiting the search to main databases and the published literature, and having one reviewer extract data and another verify (Haby et al., 2016; Tricco et al., 2015).

### Criteria for considering studies for this review

#### Population

We included studies that recruited as participants community‐dwelling children or adults (as opposed to qualified or trainee health care professionals in hospital or care home settings) across any type of study setting (e.g., schools, primary care).

#### Intervention

We included reports of evaluations of any type of intervention (e.g., mass media, face‐to‐face, technology‐mediated) designed to change at least one of six personal protective behaviours to block transmission of respiratory viruses, such as influenza, respiratory syncytial virus, parainfluenza, rhinovirus, coronavirus, or adenovirus, which have a shared route of transmission (i.e., droplets, aerosols, direct physical contact) (Killingley & Nguyen‐Van‐Tam, 2013). Although the relative importance of different personal protective behaviours depends on properties of the specific respiratory virus in addition to the clinical and/or environmental context – for example, fomite transmission may be more pronounced for respiratory syncytial virus compared with coronaviruses (Boone & Gerba, 2007) – at the time of planning this rapid review, little was known about the properties of SARS‐CoV‐2, and we therefore opted for an inclusive scope.

#### Comparison

We included studies with an active or passive (e.g., wait‐list control, baseline) comparator.

#### Outcomes

We included studies that reported as outcome either the acceptability, practicability, effectiveness, affordability, spill‐over effects, and/or equity of interventions, provided that these were measured at the individual level via self‐report or direct observation.

#### Study designs

We included primary research studies that used experimental (e.g., randomized controlled trial) or quasi‐experimental (e.g., pre‐ and post‐test) study designs, with individuals or clusters as the unit of randomization, providing that they were conducted under free‐living (as opposed to laboratory) conditions. In line with rapid review guidelines, we only included studies that were published in peer‐reviewed journals and written in English (Tricco et al., 2015; World Health Organization, 2017).

### Search methods for identification of studies

#### Electronic searches

We searched Ovid MEDLINE and Scopus. Search terms for each behaviour (e.g., ‘hand hygiene’, ‘hand washing’, ‘face mask’) were piloted and refined to achieve balance between sensitivity and specificity (see Appendix S1).

#### Searching for other sources

Expertise within the review team and consultation with topic experts was used to identify additional articles of interest. We had specified the use of reference chaining in the review protocol; however, given the rapid focus of the review and large number of identified studies, reference chaining was not performed.

### Data collection and analysis

#### Selection of studies

Two reviewers (OP and DS) independently screened (1) titles and abstracts and (2) full texts against the inclusion criteria. Discrepancies were discussed and resolved through consulting with a third reviewer (EC) if necessary.

#### Data extraction and management

A data extraction form was developed on the basis of the Behaviour Change Intervention Ontology (www.humanbehaviourchange.org) and Cochrane’s PICO ontology (https://linkeddata.cochrane.org/pico‐ontology). Ontologies are classification systems which enable researchers to specify entities (e.g., behaviours, interventions) and their inter‐relationships. The use of ontologies in systematic reviews can help ensure that a comprehensive set of entities are considered and defined in standardized ways, thus facilitating systematic knowledge synthesis (Norris, Finnerty, Hastings, Stokes, & Michie, 2019). We extracted data from relevant sections of published articles and available Appendix S1 on study design, intervention content (i.e., behaviour change techniques (BCTs), coded against the BCT Taxonomy v1 (Michie et al., 2013)), mode of delivery, population, setting, and mechanism(s) of action (Carey et al., 2019; Moore & Evans, 2017). As a validated taxonomy of mechanisms of action is, to our knowledge, not yet available, authors’ own definitions of mechanisms of action were extracted if they explicitly discussed how the selected intervention components/BCTs were expected to influence the target behaviour. Due to limited resources, we did not contact study authors for more detail on intervention descriptions. As an intervention may be effective but have negative spill‐over effects to other behaviours, or be impracticable and/or unacceptable to key stakeholders, we also deemed it important to extract data from relevant sections of published articles and available supplementary materials pertaining to the APEASE criteria (see Table 1) (Michie et al., 2014). Although criteria such as affordability or practicability are arguably closely tied to the context in which the intervention was/will be implemented, APEASE intends to capture higher‐order criteria against which to rate interventions, broadly applicable irrespective of the specific context. Therefore, authors’ own descriptions of, for example, acceptability or spill‐over effects (as opposed to reviewers’ ratings) were extracted. Spill‐over effects were broadly defined as any unintended consequences (positive or negative) reported by the authors, including but not limited to other behaviours that were changed by the intervention that it was not designed to target (e.g., teachers’ improved hand hygiene positively or negatively influencing children’s hand hygiene). Data were extracted by one reviewer (OP or DS). In the review protocol, we had specified that extracted data would be verified by a second reviewer to assess accuracy and completeness. However, given the large number of identified studies, a second reviewer (EC) verified 10% of studies.

**Table 1:** APEASE criteria for evaluating intervention approaches or components (Michie et al., 2014)

Criterion	To what extent …
Acceptability	…is the intervention judged to be acceptable by all key stakeholders
Practicability	…can the intervention be delivered as intended at the scale intended and in the context intended
Effectiveness	…will the intervention deliver the desired outcome in the target population
Affordability	…can the intervention be afforded within an acceptable budget
Spill‐over effects	…is the intervention likely to have additional negative or positive consequences
Equity	…is the intervention likely to increase or decrease inequalities in society

#### Quality appraisal

The methodological rigour of included evaluation reports was assessed by one reviewer (OP or DS) using Cochrane’s risk‐of‐bias tool (The Cochrane Collaboration, 2011). A second reviewer (EC) verified 10% of studies.

#### Stakeholder involvement

We solicited input from key stakeholders, including patient and public representatives recruited via panels convened by Public Health England (*n *= 282) and the University of East Anglia (*n* = 3), and UK policymakers and academic researchers contacted via a mailing list on the research objectives, target behaviours, and outcomes assessed. Feedback from patient and public representatives (*n* = 20) was incorporated into the review protocol; we did not receive any suggestions for improvement or clarification from the policymakers and academic researchers. The rapid review results will be disseminated to stakeholders via an infographic.

#### Data synthesis

A narrative (descriptive) synthesis was conducted for each of the personal protective behaviours. We had specified in the review protocol that meta‐analyses would be conducted if practicable and appropriate (i.e., >5 studies with homogeneous study designs and outcome variables). After inspection of study designs and outcome variables, however, we deemed it useful to conduct a meta‐analysis with *k* = 4 studies. Random‐effects meta‐analyses to estimate a pooled odds ratio (OR) or standardized mean difference (*d*) were conducted in RStudio v.1.2.5033 with the *metafor* package (Viechtbauer, 2010). Cohen’s conventions for small (*d* = .2), medium (*d* = .5), and large (*d* = .8) effects were used in the interpretation of the results (Cohen, 1988). In studies with more than two arms (e.g., three‐arm RCTs), we compared the ‘most active’ (i.e., the arm with the greatest number of intervention components) and the ‘most passive’ arms (i.e., the arm with the lowest number of intervention components, typically labelled the ‘control’ arm by study authors). Where studies reported more than one hand hygiene outcome (e.g., hand washing and hand sanitizer use), only the first reported outcome was included in the meta‐analysis, so as not to violate the assumption of independence (Cheung, 2019). Where studies did not report sufficient detail to calculate effect sizes, authors’ own description/interpretation of results were grouped into ‘positive’ effects (i.e., a significant difference between intervention and control groups, favouring the intervention group, was detected), ‘no difference’ (i.e., a significant difference between groups was not detected), ‘negative’ effects (i.e., a significant difference between groups, favouring the control group, was detected), or ‘indeterminate’ (i.e., differences between groups were not reported or could not be computed given the study design). To aid interpretation, for behaviours where a majority of positive or negative results were observed, overall results were categorized as either ‘positive’ or ‘negative’, respectively. If consistent results were not observed or could not be determined, overall results were categorized as ‘mixed’.

## Results

### Study selection

**Figure 2:**
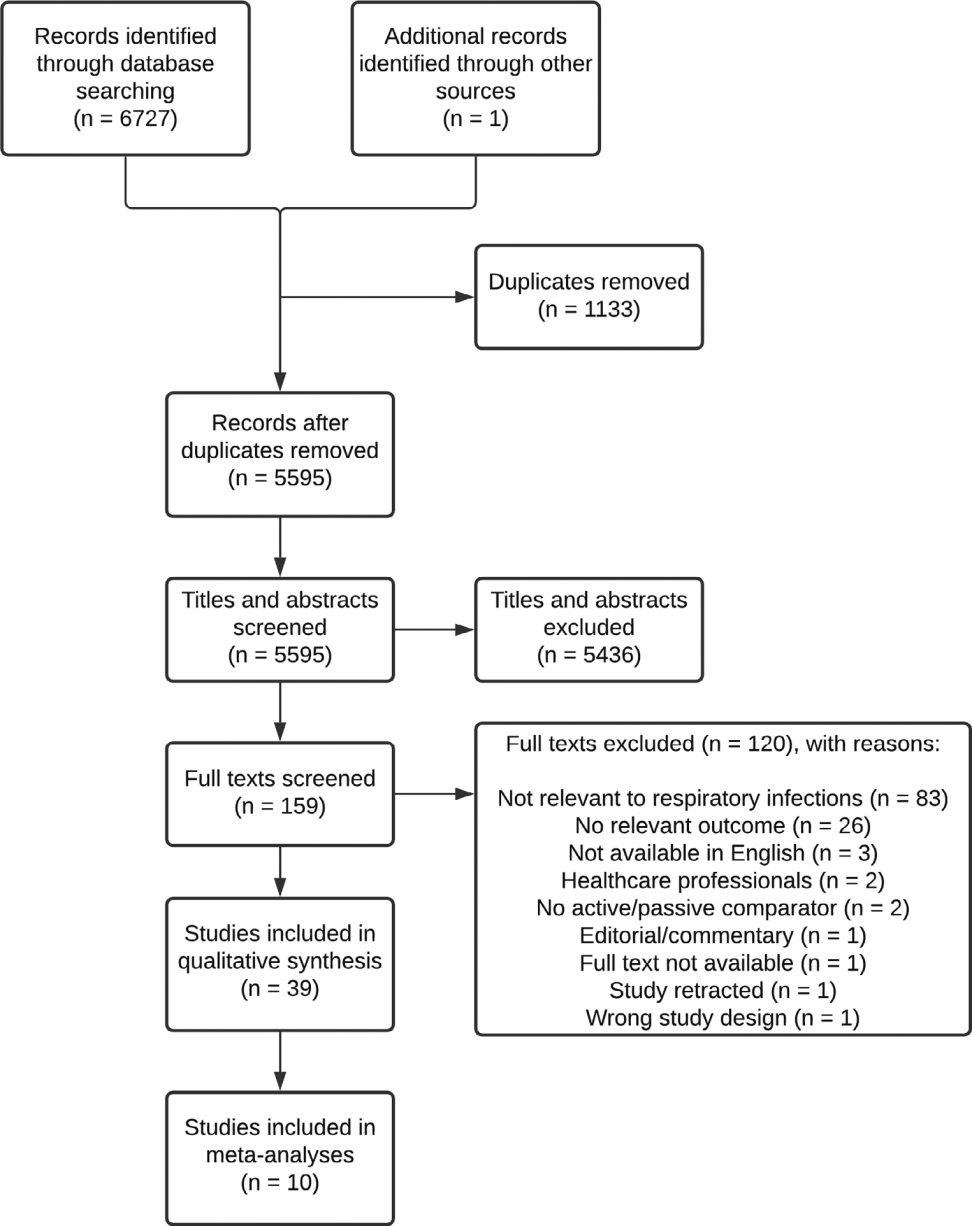
PRISMA flow chart of included studies.

After removing duplicates, 5,595 records were identified, with 159 studies carried forward to the full text screening. Of the 39 studies included in the narrative evidence synthesis, 10 were included in meta‐analyses (see Figure 2).

### Study characteristics

Studies were conducted in the United States (13/39; 33%) (Aiello et al., 2010, 2012; Arbogast et al., 2016; Azman et al., 2013; Koep et al., 2016; Larson, Ferng, McLoughlin, Wang, & Morse, 2009; Larson et al., 2010; Mott et al., 2007; Sandora et al., 2005; Stebbins, Stark, & Vukotich, 2010; Stedman‐Smith et al., 2015; Updegraff, Emanuel, Gallagher, & Steinman, 2011; White, Kolble, Carlson, Lipson, & Dolan, 2003), China (5/39; 13%) (Chan, So, Wong, Lee, & Tiwari, 2007; Cowling et al., 2008, 2009; Liu et al., 2019; Or, Ching, & Chung, 2020), Germany (3/39; 13%) (Hübner, Hübner, Wodny, Kampf, & Kramer, 2010; Suess et al., 2011, 2012), Thailand (3/39; 13%) (Apisarnthanarak, Apisarnthanarak, Cheevakumjorn, & Mundy, 2009; Kaewchana et al., 2012; Simmerman et al., 2011), Australia (2/39; 5%) (MacIntyre et al., 2009; Roberts et al., 2000), Denmark (2/39; 5%) (Bundgaard et al., 2020; Nandrup‐Bus, 2009), Spain (2/39; 5%) (Azor‐Martinez et al., 2016, 2018), and the United Kingdom (2/39; 5%) (Little et al., 2015; Yardley, Miller, Schlotz, & Little, 2011), with one study each in Australia/Saudi Arabia (Barasheed et al., 2014), Bangladesh (Ram et al., 2015), Costa Rica (Reyes Fernández, Lippke, Knoll, Moya, & Schwarzer, 2015), Finland (Savolainen‐Kopra et al., 2012), France (Canini et al., 2010), the Netherlands (Zomer et al., 2016), and Turkey (Öncü et al., 2019) (see Table 2). Studies had a median of 419 participants (range: 96–20,066).

**Table 2:** Characteristics of included studies

Lead author (year)	Country	Respiratory virus targeted	Target behaviour(s)	Study design	Population	Sample size	Mean age (*SD*)	% Female	% Post‐16 educational qualifications	Setting	Recruitment strategy
(1) Aiello (2010)	United States	Pandemic influenza A (H1N1)	Face mask use and hand hygiene	Three‐arm, cluster RCT	University students in residence halls	1,297 (face mask + hand hygiene = 367; face mask = 378; control = 552)	18.7 (0.8)	66%	100%	University residence halls	Not reported
(2) Aiello (2012)	United States	Influenza A or B	Face mask use and hand hygiene	Three‐arm, cluster RCT	University students in residence halls	1178 (face mask + hand hygiene = 362; face mask = 420; control = 396)	19.0 (0.9)	55%	100%	University residence halls	Not reported
(3) Apisarnthanarak (2009)	Thailand	Influenza	Hand hygiene	Single arm, pre‐ and post‐intervention study	Preschool children	240	5.0 (1.7)	49%	0%	Private kindergarten	Not reported
(4) Arbogast (2016)	United States	Respiratory syncytial virus, adenovirus, influenza	Hand hygiene	Two‐arm, cluster RCT	Office workers	1,386 (intervention = 604; control = 782)	47.0 (0.4)	78%	Not reported	Office buildings	E‐mail sent to employees
(5) Azman (2013)	United States	Influenza A or B	Hand hygiene	Two‐arm, cluster RCT	Households	3,360	Not reported	Not reported	0%	Schools	Not reported
(6) Azor‐Martinez (2016)	Spain	Influenza	Hand hygiene	Two‐arm RCT	School children	1,341	8.0 (2.3)	68%	0%	Schools	Not reported
(7) Azor‐Martinez (2018)	Spain	Respiratory viral infections	Hand hygiene	Three‐arm, cluster RCT	Households with a child at a daycare centre	911 children	Unclear	Unclear	Unclear	Daycare centres	Not reported
(8) Barasheed (2014)	Australia/Saudi Arabia	Rhinovirus, influenza, parainfluenza	Face mask use	Two‐arm, pilot RCT	Hajj pilgrims	164	Not reported	Not reported	Not reported	Not reported	Study brochures distributed in mosques, Islamic centres and pre‐travel seminars, and in hotels in Mecca
(9) Bundgaard (2020)	Denmark	SARS‐CoV‐2	Face mask use	Two‐arm RCT	Community‐dwelling adults	6,304	47.2 (13.5)	64%	Not reported	Participants’ own homes	Media advertisements and through contacting private companies and public organizations
(10) Canini (2010)	France	Influenza	Face mask use	Two‐arm, cluster RCT	Households in three regions of France with an index patient	105	26.5 (16.0)	Not reported	Not reported	GP offices	GPs
(11) Chan (2007)	China	SARS	Catching droplets in tissues and hand hygiene	Single arm, pre‐ and post‐intervention study	Registered members of a government subsidized social service centre	122	Not reported	63%	20%	Participants’ own homes	Telephone calls
(12) Cowling (2008)	China	Influenza A or B	Face mask use and hand hygiene	Three‐arm, cluster RCT	Households with an index patient	198	Not reported	56%	Not reported	Participants’ own homes	Outpatient clinics
(13) Cowling (2009)	China	Influenza A or B	Face mask use and hand hygiene	Three‐arm, cluster RCT	Households with an index patient	407 index patients	Not reported	51%	Not reported	Participants’ own homes	Outpatient clinics
(14) Hübner (2010)	Germany	Common cold, influenza	Hand hygiene	Two‐arm RCT	Office workers	134	44.6 (–)	86%	Not reported	Office buildings	E‐mails
(15) Kaewchana (2012)	Thailand	Influenza	Hand hygiene	Two‐arm RCT	Households with an index paediatric patient	275 (frequency assessment) + 330 (quality assessment) households	34.7 (13.8)	58%	Not reported	Participants’ own homes	Not reported
(16) Koep (2016)	United States	Influenza	Hand hygiene	Two‐arm, non‐randomized cohort study	School children	260	Not reported	46%	0%	Schools	Not reported
(17) Larson (2009)	United States	Rhinovirus, coronavirus, parainfluenza virus, respiratory syncytial virus, adenovirus, influenza, enterovirus, etc.	Hand hygiene	Single arm, pre‐ and post‐intervention study	Households	422 households	Not reported	Not reported	60%	Participants’ own homes	Neighbourhood snowballing techniques (e.g., churches, schools, clinics)
(18) Larson (2010)	United States	Influenza, respiratory syncytial virus, parainfluenza, enterovirus, rhinovirus, adenovirus, metapneumovirus	Face mask use and hand hygiene	Three‐arm RCT	Households	509	Not reported	52%	54%	Participants’ own homes	Neighbourhood snowballing techniques (e.g., churches, schools, clinics)
(19) Little (2015)	United Kingdom	Influenza	Hand hygiene	Two‐arm RCT	Community‐dwelling adults	20,066	56.6 (13.6)	56%	Not reported	Online	Mailed invitations through GP surgeries
(20) Liu (2019)	China	Respiratory viral infections	Hand hygiene	Single arm, pre‐ and post‐intervention study	Kindergarten teachers	361	29.0 (8.7)	95%	Not reported	Schools	Not reported
(21) MacIntyre (2009)	Australia	Influenza, respiratory syncytial virus, adenovirus, parainfluenza virus, coronavirus, metapneumovirus	Face mask use and hand hygiene	Three‐arm, cluster RCT	Households with an index paediatric patient	145 families, 290 caregivers	Not reported	Not reported	Not reported	Not reported	Paediatric clinic waiting rooms
(22) Mott (2007)	United States	Respiratory illnesses (e.g., flu, colds)	Hand hygiene	Three‐arm, non‐randomized cohort study	Military trainees	2,728	20.2 (–)	0%	Unclear	US army training centre	Not reported
(23) Nandrup‐Bus (2020)	Denmark	Respiratory infections	Hand hygiene	Two‐arm, cluster RCT	School children	652	Not reported	51%	0%	Schools	Parents were sent written study information
(24) Or (2020)	China	Influenza	Hand hygiene	Single arm, pre‐ and post‐intervention study	Households with a child in kindergarten	58 parents; 60 children	Not reported	Parents = 93%; children = 40%	Not reported	Not reported	Letters sent to principals of 15 kindergartens to invite parents and children to participate
(25) Ram (2015)	Bangladesh	Influenza	Hand hygiene	Two‐arm, cluster RCT	Households	377 index cases; 384 household compounds	121.2 months (181.7)	40%	Not reported	Participants’ own homes	Outpatient clinics
(26) Reyes Fernández (2015)	Costa Rica	Respiratory infections	Hand hygiene	Two‐arm, cluster RCT	University students	242	21.0 (3.9)	61%	100%	University classrooms	Not reported
(27) Roberts (2000)	Australia	Respiratory infections	Hand hygiene	Two‐arm, cluster RCT	Preschool children and daycare staff	232 childcare centres; 558 children	Not reported	Not reported	0%	Childcare centres	Directors of daycare centres were invited
(28) Sandora (2005)	United States	Respiratory infections	Hand hygiene	Two‐arm, cluster RCT	Households with a child at a daycare centre	292 families	36.7 (9.5)	Not reported	91%	Participants’ own homes	Directors of daycare centres were invited. A recruitment letter was sent to parents.
(29) Savolainen‐Kopra (2012)	Finland	Respiratory infections	Hand hygiene	Three‐arm, cluster RCT	Office workers	683	Not reported	Not reported	Not reported	Office buildings	E‐mails
(30) Simmerman (2011)	Thailand	Influenza	Face mask use and hand hygiene	Three‐arm RCT	Households	442 index cases; 1,147 household contacts	Not reported	59%	Not reported	Participants’ own homes	Outpatient clinics
(31) Stebbins (2010)	United States	Influenza and related respiratory infections	Hand hygiene	Two‐arm, cluster RCT	School children and teachers	151 teachers	Not reported	Not reported	Not reported	Schools	Not reported
(32) Stedman‐Smith (2015)	United States	Respiratory infections	Hand hygiene	Two‐arm, cluster RCT	Office workers	324	Not reported	84%	100%	Office buildings	E‐mails
(33) Suess (2011)	Germany	Pandemic influenza A (H1N1)	Face mask use and hand hygiene	Three‐arm, cluster RCT	Households with an index patient during the H1N1 pandemic	147	Index cases = 7.9 (3.3); household contacts = 30.0 (14.2)	52%	Not reported	Participants’ own homes	Outpatient clinics
(34) Suess (2012)	Germany	Pandemic influenza A (H1N1)	Face mask use and hand hygiene	Three‐arm, cluster RCT	Households with an index patient during the H1N1 pandemic	302	Not reported	Not reported	Not reported	Participants’ own homes	Outpatient clinics
(35) Updegraff (2011)	United States	Pandemic influenza A (H1N1)	Hand hygiene	Randomized cross‐over study	University students and staff	65 units	Not reported	Not reported	Not reported	Public areas of a university	No active recruitment
(36) White (2003)	United States	Respiratory infections	Hand hygiene	Two‐arm, cohort study^a^	University students	430	18.3 (0.7)	62%	0%	University residence halls	Not reported
(37) Yardley (2011)	United Kingdom	Respiratory infections	Hand hygiene	Two‐arm, pilot RCT	Community‐dwelling adults	517	49.8 (11.4)	64%	Not reported	Online	Mailed invitations from GP surgeries
(38) Zomer (2016)	The Netherlands	Respiratory infections	Hand hygiene	Two‐arm, cluster RCT	Households with a child at a daycare centre	71 centres	Not reported	Not reported	Not reported	Not reported	Not reported
(39) Öncü (2019)	Turkey	Respiratory infections	Hand hygiene	Three‐arm, cluster RCT	School children	96	9.2 (1.0)	54%	0%	School laboratory	Not reported

Note

^a^Unclear if randomized.

The majority of studies (20/39; 51%) targeted multiple respiratory infections, including (but not limited to) influenza, respiratory syncytial virus, and adenovirus (Arbogast et al., 2016; Azor‐Martinez et al., 2018; Barasheed et al., 2014; Hübner, Hübner, Wodny, Kampf, & Kramer, 2010; Larson et al., 2009; Larson et al., 2010; Liu et al., 2019; MacIntyre et al., 2009; Mott et al., 2007, p. 20; Nandrup‐Bus, 2020; Öncü et al., 2019; Reyes Fernández, Lippke, Knoll, Moya, & Schwarzer, 2015; Roberts et al., 2000; Sandora et al., 2005; Savolainen‐Kopra et al., 2012; Stebbins et al., 2010; Stedman‐Smith et al., 2015, p.; White et al., 2003; Yardley et al., 2011; Zomer et al., 2016), with the remaining studies targeting influenza (13/39; 33%) (Aiello et al., 2012; Apisarnthanarak et al., 2009; Azman et al., 2013; Azor‐Martinez et al., 2016; Canini et al., 2010; Cowling et al., 2008, 2009; Kaewchana et al., 2012; Koep et al., 2016; Little et al., 2015; Or et al., 2020; Ram et al., 2015; Simmerman et al., 2011), pandemic influenza A (H1N1) (4/39; 10%) (Aiello et al., 2010; Suess et al., 2011, 2012; Updegraff et al., 2011), SARS (1/39; 3%) (Chan et al., 2007), and SARS‐CoV‐2 (1/39; 3%) (Bundgaard et al., 2020) (see Table 2).

The majority of studies (28/39; 72%) targeted hand hygiene, with the remaining studies targeting a combination of hand hygiene and/or face mask use (9/39; 23%) (Aiello et al., 2010, 2012; Cowling et al., 2008, 2009; Larson et al., 2010; MacIntyre et al., 2009; Simmerman et al., 2011; Suess et al., 2011, 2012), face mask use only (3/39; 8%) (Barasheed et al., 2014; Bundgaard et al., 2020; Canini et al., 2010), or a combination of catching of droplets in tissues and hand hygiene (1/39; 3%) (Chan et al., 2007) (see Table 2). Interventions were delivered in participants’ own homes (12/39; 31%), nurseries/schools (10/39; 26%), university residence halls/public areas (5/39; 13%), offices (4/39; 10%), online (2/39; 5%), outpatient clinics (1/39; 3%), or an army training centre (1/39; 3%). Four studies did not state the setting for intervention delivery. Studies targeted children and adult household members (16/39; 41%), pre‐ or school children and/or teachers (8/39; 21%), university staff and/or students (5/39; 13%), office workers (4/39; 10%), community‐dwelling adults (3/39; 8%), Hajj pilgrims (1/39; 3%), military trainees (1/39; 3%), or adult members of a social service centre (1/39; 3%).

Study designs used were two‐arm, cluster RCTs (11/39; 28%), three‐arm, cluster RCTs (10/39; 26%), two‐arm RCTs (5/39; 13%), single‐arm, pre‐ and post‐intervention studies (5/39; 13%), two‐arm, non‐randomized cohort studies (2/39; 5%), three‐arm RCTs (2/39; 5%), two‐arm, pilot RCTs (1/39; 3%), or randomized, cross‐over studies (1/30; 3%).

### Intervention characteristics

Intervention durations ranged from one day (i.e., one‐off interventions) to 3 years (see Table 3). In studies using a two‐ or three‐arm design (33/39; 85%), comparators included no intervention/usual care (19/33; 58%), educational materials (9/33; 27%), the provision of soap/hand sanitizer (3/33; 9%), or a combination of educational materials and soap/hand sanitizer (2/33; 6%). Interventions were delivered via face‐to‐face sessions (23/39; 59%), written materials (including books and newsletters) (14/39; 36%), posters/bulletin boards/cue cards (8/39; 21%), cartoons/games (4/39; 10%), videos (4/39; 10%), telephone (3/39; 8%), and/or websites (2/39; 5%). Five studies did not clearly report on the mode of intervention delivery. Where reported, participants received a flat payment for study completion (Aiello et al., 2010; Cowling et al., 2008, 2009; Simmerman et al., 2011; Suess et al., 2011, 2012; White et al., 2003), payment per survey completed (Stebbins et al., 2010), points for study completion (Arbogast et al., 2016), or points per survey completed (Stedman‐Smith et al., 2015).

**Table 3:** Characteristics of interventions to change personal protective behaviours

Lead author (year)	Comparator	Intervention	Intervention duration	BCTs (comparator arm)	BCTs (intervention arm)	Intervention mode of delivery	Theoretical mechanism(s) of action of the intervention	Incentive structure for study participation
(1) Aiello (2010)	Educational materials	The face mask group received face masks; written instructions on how to use, store, and safely discard masks. The face mask + hand hygiene group also received hand sanitizer	6 weeks	4.1. Instruction on how to perform the behaviour	4.1. Instruction on how to perform the behaviour; 6.1. Demonstration of the behaviour; 8.1. Behaviour practice/rehearsal; 12.5. Adding objects to the environment	Video link, written materials	Not reported	Those with influenza‐like illness were offered $25 for providing a throat specimen
(2) Aiello (2012)	Educational materials	The face mask group received face masks; written instructions on how to use, store, and safely discard masks. The face mask + hand hygiene group also received hand sanitizer	6 weeks	4.1. Instruction on how to perform the behaviour	4.1. Instruction on how to perform the behaviour; 8.1. Behaviour practice/rehearsal; 12.5. Adding objects to the environment	Written materials	Not reported	Not reported
(3) Apisarnthanarak (2009)	NA	Children, teachers and parents received hand hygiene (including sanitizer) education via cartoons and workshops. A single dispenser of alcohol‐based hand rub was placed in each nursery room	3 years	NA	2.2 Feedback on behaviour; 2.7 Feedback on outcomes of behaviour; 4.1. Instruction on how to perform the behaviour; 6.1. Demonstration of the behaviour; 8.1. Behaviour practice/rehearsal; 12.5. Adding objects to the environment	Face‐to‐face workshop, cartoons, written materials	Not reported	Not reported
(4) Arbogast (2016)	An educational video about hand hygiene; soap and hand sanitizer provided in toilets	Same as the control group in addition to hand sanitizers being provided in different areas of the office building	13.5 months	4.1. Instruction on how to perform the behaviour; 12.5. Adding objects to the environment	4.1. Instruction on how to perform the behaviour; 6.1. Demonstration of the behaviour; 12.5. Adding objects to the environment	Videos	Not reported	25 ‘wellness points’ were offered to employees who completed both baseline and post‐study survey
(5) Azman (2013)	Hand sanitizer	Same as control group in addition to a live demonstration of hand washing behaviour. Information about hand hygiene was sent home	Not reported	12.5. Adding objects to the environment	4.1. Instruction on how to perform the behaviour; 6.1. Demonstration of the behaviour; 12.5. Adding objects to the environment	Face‐to‐face workshop, written materials	Not reported	Not reported
(6) Azor‐Martinez (2016)	No intervention	Handwashing workshop; hand hygiene practices were periodically reinforced in the classroom; younger children were supervised during hand hygiene procedures; provision of hand sanitizer	8 months	NA	4.1. Instruction on how to perform the behaviour; 6.1. Demonstration of the behaviour; 8.1. Behaviour practice/rehearsal; 12.5. Adding objects to the environment	Face‐to‐face workshop	Not reported	Not reported
(7) Azor‐Martinez (2018)	No intervention	Hand hygiene workshop with instruction on how to correctly perform the behaviour; use of stories, songs, posters; provision of hand sanitizer in one group and liquid soap in the other group; written materials on hand hygiene	8 months	NA	4.1. Instruction on how to perform the behaviour; 6.1. Demonstration of the behaviour; 8.1. Behaviour practice/rehearsal; 12.5. Adding objects to the environment	Face‐to‐face workshop, written materials	Not reported	Not reported
(8) Barasheed (2014)	Hygiene information	In addition to the general hygiene information, face masks and written and verbal instructions on how to use these were provided	5 days	Not reported	4.1. Instruction on how to perform the behaviour; 12.5. Adding objects to the environment	Face‐to‐face instructions, written materials	Not reported	Not reported
(9) Bundgaard (2020)	Weekly e‐mails encouraging participants to follow current COVID‐19 recommendations	Instructions to wear a mask when outside the home during the next month; provision of 50 three‐layer, disposable, surgical face masks with ear loops	1 month	NA	4.1 Instruction on how to perform the behaviour; 12.5 Adding objects to the environment	Written materials	Not reported	Not reported
(10) Canini (2010)	No intervention	Provision of face masks and demonstration of how to use them	3 weeks	NA	6.1. Demonstration of the behaviour; 12.5. Adding objects to the environment	Face‐to‐face instructions	Not reported	Not reported
(11) Chan (2007)	NA	Health education	7 days	NA	Not reported	Telephone calls with trained nursing students	Not reported	Not reported
(12) Cowling (2008)	No intervention (education about healthy diet and lifestyle)	The face mask group received face masks, information about the efficacy of masks and instruction on how to use and safely dispose of masks; the hand hygiene group received hand sanitizer, liquid soap, information about the efficacy of hand hygiene and demonstration of hand hygiene behaviour	9 days	NA	4.1. Instruction on how to perform the behaviour; 5.1. Information about health consequences; 6.1. Demonstration of the behaviour; 12.5. Adding objects to the environment;	Not reported	Not reported	At the final home visit, households were reimbursed for their participation with a supermarket voucher worth approximately US $20
(13) Cowling (2009)	No intervention (education about healthy diet and lifestyle)	Hand hygiene; surgical face masks plus hand hygiene	6 days	NA	5.1. Information about health consequences; 12.5. Adding objects to the environment	Not reported	Not reported	At the final home visit, households were reimbursed for their participation with a supermarket voucher worth approximately US $25
(14) Hübner (2010)	No intervention	Provision of hand sanitizer and instruction on how and when to use it at work	12 months	NA	4.1. Instruction on how to perform the behaviour; 12.5. Adding objects to the environment	Not reported	Not reported	Not reported
(15) Kaewchana (2012)	30‐minute routine health education on influenza infection, nutrition, physical activity, and smoking cessation	30‐minute intensive hand washing education; individual training on hand washing; provision of liquid soap; self‐monitoring diary; written materials on hand washing techniques	3 months	4.1. Instruction on how to perform the behaviour; 5.1. Information about health consequences	2.3. Self‐monitoring of behaviour; 4.1. Instruction on how to perform the behaviour; 5.1. Information about health consequences; 5.3. Information about social and environmental consequences; 8.1. Behaviour practice/rehearsal; 12.5. Adding objects to the environment	Face‐to‐face workshop	Not reported	Not reported
(16) Koep (2016)	No intervention	Educational intervention with information about microorganisms; provision of liquid soap and hand sanitizer in toilets and classrooms	Unclear	NA	4.1. Instruction on how to perform the behaviour; 5.1. Information about health consequences; 12.5. Adding objects to the environment	Face‐to‐face with trained teachers	Not reported	Not reported
(17) Larson (2009)	NA	Educational intervention focused on infection control; question and answer fact sheets; two groups of households were randomized to receive hand sanitizer, face masks, or both	2 to 20 months	NA	4.1. Instruction of how to perform the behaviour; 5.1. Information about health consequences; 8.1. Behaviour practice/rehearsal; 12.5. Adding objects to the environment	Face‐to‐face with trained researchers	Not reported	Not reported
(18) Larson (2010)	Educational materials on infection control	The hand sanitizer group received education plus hand sanitizer; the hand sanitizer and face mask group received the same interventions plus face masks	19 months	Not reported	4.1. Instruction on how to perform the behaviour; 5.1. Information about health consequences; 8.1. Behaviour practice/rehearsal; 12.5. Adding objects to the environment	Face‐to‐face with trained researchers	Not reported	Not reported
(19) Little (2015)	No intervention	Four weekly web‐based sessions with new content focused on the role of hand washing, setting up a plan to wash hands, reinforcement of helpful attitudes and norms, addressing negative beliefs, tailored feedback and prompts to login to the website	4 months	NA	1.4. Action planning; 2.2. Feedback on behaviour; 4.1. Instruction on how to perform the behaviour; 7.1 Prompts/cues; 8.1. Behaviour practice/rehearsal	Website	Not reported	Not reported
(20) Liu (2019)	NA	Hand hygiene training and information booklet; provision of soap, towels, posters, stickers, books, memory games, and diplomas	6 months	NA	4.1 Instruction on how to perform the behaviour; 10.4 Social reward; 12.5 Adding objects to the environment	Face‐to‐face sessions, posters, stickers, books	Knowledge, perceived susceptibility, perceived severity, perceived behavioural control	Not reported
(21) MacIntyre (2009)	No intervention	Information about infection control; provision of either P2 or surgical face masks	Unclear	NA	12.5 Adding objects to the environment	Not reported	Not reported	Not reported
(22) Mott (2007)	Hand sanitizer and instructions to wash or sanitize hands after key events (e.g., coughing, sneezing)	In the PI group, hand sanitizer dispensers were installed throughout the training environment; provision of personal hand sanitizer bottles; posters were placed in training facilities to encourage hand hygiene and foster a sense of pride in staying healthy; instruction on hand hygiene; and weekly reminders by drill sergeants to carry, use, and refill hand sanitizer bottles. The SI group received personal hand sanitizer bottles and instruction on hand hygiene only	Not reported	12.5 Adding objects to the environment	4.1 Instruction on how to perform the behaviour; 9.1 Credible source; 12.5 Adding objects to the environment	Face‐to‐face sessions, posters	Not reported	Not reported
(23) Nandrup‐Bus (2020)	NA	Children were required to wash their hands three times per day; training on hand washing and infection control was provided; posters with step‐by‐step hand washing instructions placed by wash basins; parents were asked to remind children to wash their hands before the first lesson each day	3 months	NA	4.1 Instruction on how to perform the behaviour; 5.1 Information about health consequences	Face‐to‐face sessions, posters	Not reported	Not reported
(24) Or (2020)	No intervention	Children attended 4 weekly sessions with information about infection control and hand hygiene techniques; parents attended a separate session with similar content	4 weeks	NA	4.1 Instruction on how to perform the behaviour; 5.1 Information about health consequences	Face‐to‐face sessions with an infection control nurse	Knowledge, skills	Not reported
(25) Ram (2015)	No intervention	Household compounds were provided with a hand washing station (e.g., water container with a tap, soap); information on infection control and skills training; cue cards placed in a common area in compound courtyards	Tailored; daily intervention visits until 10 days following the resolution of the index case patient's symptoms	NA	4.1 Instruction on how to perform the behaviour; 5.1 Information about health consequences; 12.5 Adding objects to the environment	Face‐to‐face sessions, cue cards	Not reported	Not reported
(26) Reyes Fernández (2015)	No intervention	Instructions on how and when to clean hands and a planning task to help students set action and coping plans	One‐off	NA	1.2 Problem solving; 1.4 Action planning; 4.1 Instruction on how to perform the behaviour	A face‐to‐face session with research assistants, pamphlets	Action control, coping planning	Not reported
(27) Roberts (2000)	No intervention	Staff received training in hand washing and were asked to teach the techniques to the children in their care via songs about hand washing to the melodies of nursery rhymes; training was reinforced with fortnightly visits and newsletters	Not reported	NA	4.1 Instruction on how to perform the behaviour; 6.1 Demonstration of the behaviour	Face‐to‐face training sessions, newsletters	Not reported	Not reported
(28) Sandora (2005)	No intervention (educational materials about healthy eating; participants were asked not to use hand sanitizer during the study period)	Provision of alcohol‐based hand sanitizer; educational materials (e.g., fact sheets, games, toys) about hand hygiene	5 months	NA	12.5 Adding objects to the environment	Fact sheets, games, toys	Not reported	Not reported
(29) Savolainen‐Kopra (2012)	No intervention	Both groups received information on infection control. In the soap and water group, toilets were equipped with liquid hand soap. In the hand sanitizer arm, toilets were equipped with both liquid hand soap and alcohol‐based hand rub	18 months	NA	4.1 Instruction on how to perform the behaviour; 5.1 Information about health consequences; 12.5 Adding objects to the environment	Not reported	Not reported	Not reported
(30) Simmerman (2011)	No intervention (nutritional, physical activity, and smoking cessation education)	The hand washing group received education, instruction on hand washing techniques and a hand washing kit with liquid hand soap. The hand washing + face mask group received the same interventions as the hand washing group in addition to paper surgical face masks, training on how to use them appropriately and information about benefits of use	7 days	NA	4.1 Instruction on how to perform the behaviour; 5.1 Information about health consequences; 12.5 Adding objects to the environment	Face‐to‐face sessions with trained study nurses	Not reported	Households were compensated with approximately US $60 in Thai baht
(31) Stebbins (2010)	No intervention	Students and staff received training in hand hygiene behaviours; schools placed and maintained supplies of alcohol‐based hand sanitizer in all classrooms and common areas; parents and guardians received educational materials on hand hygiene and home isolation practices	Not reported	NA	4.1 Instruction on how to perform the behaviour; 5.1 Information about health consequences; 12.5 Adding objects to the environment	Face‐to‐face sessions, videos	Not reported	Teachers were offered a $5 gift card for completion of each survey
(32) Stedman‐Smith (2015)	A brief training video to promote more effective communication with healthcare providers, branded key chains, brochures and posters with information about the programme	A brief training video on infection control and demonstration of effective hand washing/gel techniques; hand sanitizer and motivational/educational hand hygiene posters were placed in break rooms, kitchens, and conference rooms	Not reported	Not reported	4.1 Instruction on how to perform the behaviour; 12.5 Adding objects to the environment	Video, posters	Not reported	Points towards health promotion items within an employee wellness programme were provided for each survey completed
(33) Suess (2011)	Educational materials about infection control and recommendation to sleep in a different room than the index patient	Participants in the mask + hand hygiene and mask groups were provided with surgical face masks with ear loops and written information on their correct use; participants in the mask + hand hygiene group were provided with alcohol‐based hand rub and instructions on correct use	8 days	Not reported	4.1 Instruction on how to perform the behaviour; 12.5 Adding objects to the environment	Written materials, telephone, face‐to‐face visits by trained study personnel	Not reported	€150 for study participation
(34) Suess (2012)	Educational materials about infection control and recommendation to sleep in a different room than the index patient	Participants in the mask + hand hygiene and mask groups were provided with surgical face masks with ear loops and written information on their correct use; participants in the mask + hand hygiene group were provided with alcohol‐based hand rub and instructions on correct use	8 days	Not reported	4.1 Instruction on how to perform the behaviour; 6.1 Demonstration of the behaviour; 12.5 Adding objects to the environment	Written materials, telephone, face‐to‐face visits by trained study personnel	Not reported	€150 for study participation
(35) Updegraff (2011)	Hand sanitizer	Four signs were placed above hand sanitizer units. The perceived susceptibility headline read ‘Germs are out to get you. Get them first!’; the social norms headline read ‘Everybody is doing it. Are you?’; the gain‐framed headline read ‘Stay healthy this season. Sanitize your hands’; and the loss‐framed headline read ‘H1N1. Getting it is as easy as passing me by’. Each sign contained a ‘fact box’ with more detailed information reinforcing the theme	12 weeks	12.5 Adding objects to the environment	5.1 Information about health consequences; 5.2 Information about social and environmental consequences; 12.5 Adding objects to the environment	Foam boards	Perceived susceptibility, social norms, and attitudes towards the behaviour	Not reported
(36) White (2003)	No intervention	Alcohol‐gel dispensers were installed in every room, washroom, and dining hall in residence halls; a hand washing message campaign was implemented with bulletin boards and weekly messages to encourage hand washing	10 weeks	NA	12.5 Adding objects to the environment	Bulletin boards	Not reported	Cash incentives totalling a maximum of $65
(37) Yardley (2011)	No intervention	Four weekly web‐based sessions with information about the medical team behind the advice (to enhance credibility), infection control, expert recommendations for hand washing frequency and technique, and instructions for picking up a free supply of hand gel from one’s local GP practice; a hand washing plan to promote intention formation with situational cueing; tailored feedback to help participants improve their plan; reinforcement of positive attitudes and norms; addressing common negative beliefs; e‐mail prompts to login to the website	4 weeks	NA	1.4 Action planning; 2.2 Feedback on behaviour; 5.1 Information about health consequences; 7.1 Prompts/cues; 9.1 Credible source; 12.5 Adding objects to the environment	Website	Intention to wash hands, attitude, subjective norms, perceived behavioural control, perceived risk of infection	Not reported
(38) Zomer (2016)	Usual care	Four components: (1) free hand hygiene products with refills for 6 months (e.g., soap, hand sanitizer); (2) a handwashing exercise with 'UV Glow Cream' and an information booklet with the training content; (3) two team training sessions focused on goal setting and identifying hand hygiene improvement activities; and (4) posters and stickers placed in daycare centres acting as reminders to practise hand hygiene	6 months	Not reported	1.1 Goal setting (behaviour); 7.1 Prompts/cues; 8.1 Behavioural practice/rehearsal; 12.5 Adding objects to the environment	Face‐to‐face sessions, posters, stickers, booklets	Not reported	Not reported
(39) Öncü (2019)	Hand gel and 'photo shoots' before and after handwashing	The first group received the same intervention as the control group plus information about getting rid of microbes on hands if washing hands 'properly'; the second group received the same interventions as the first plus a 30‐minute hand hygiene educational and training session on types of microbes and diseases caused by microbes, in addition to WHO's 9‐stage handwashing programme	4 weeks	12.5 Adding objects to the environment	4.1 Instruction on how to perform the behaviour; 5.1 Information about health consequences; 8.1 Behavioural practice/rehearsal; 12.5 Adding objects to the environment	Face‐to‐face session	Not reported	Not reported

Note

NA = not applicable.

**Figure 3:**
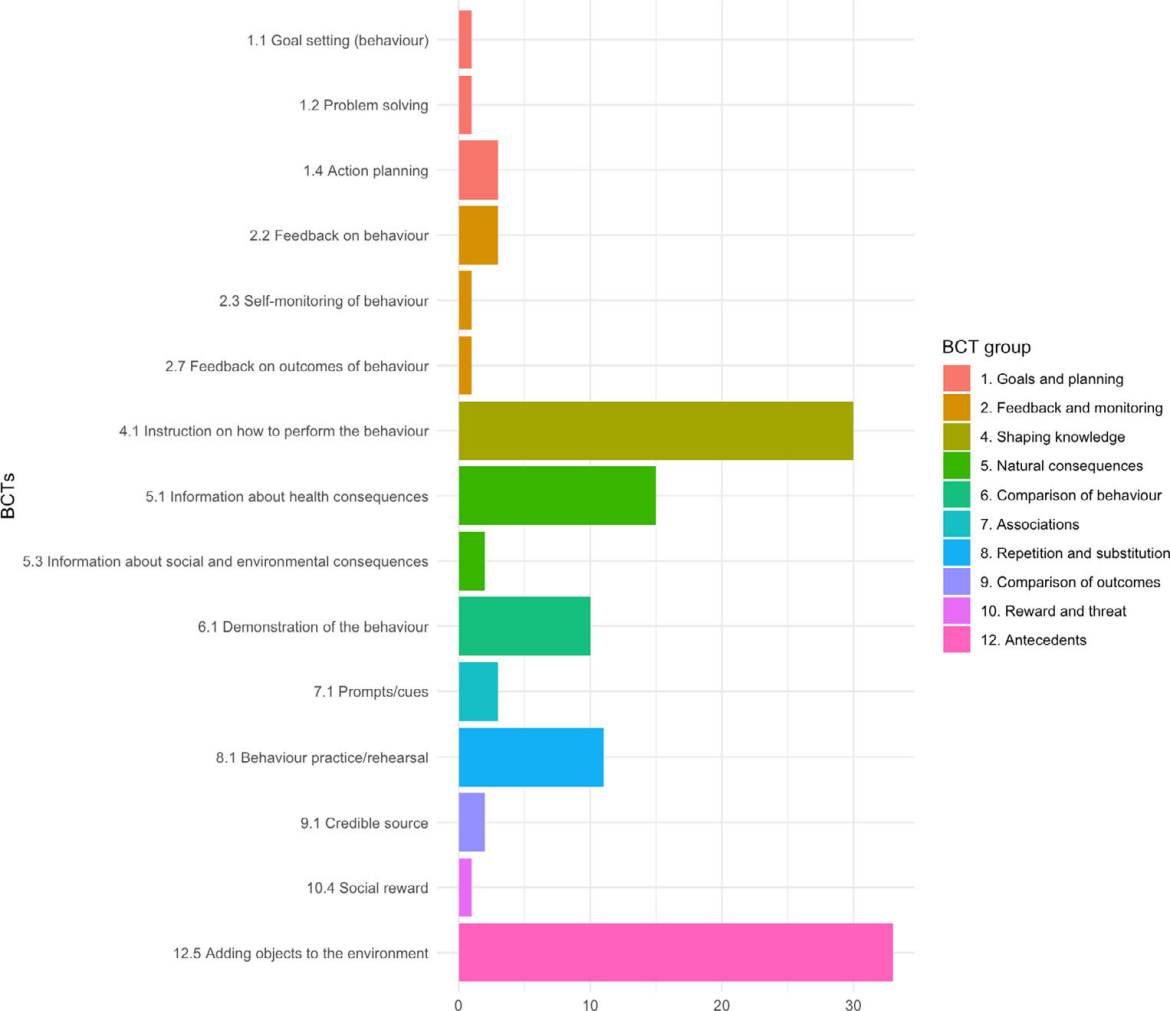
Frequencies of behaviour change techniques (BCTs) coded in published intervention descriptions.

We coded 15 different BCTs across intervention descriptions (see Figure 3); however, details on intervention content were typically lacking. Interventions included a median of three BCTs (range: 0 to 6). The most frequently coded BCTs were ‘12.5 Adding objects to the environment’ (17/39; 44%), ‘4.1 Instruction on how to perform the behaviour’ (16/39; 41%), and ‘5.1 Information about health consequences’ (10/39; 26%). Few studies reported targeting a specific mechanism of action. Where reported, interventions were designed to target intentions (Yardley et al., 2011), attitudes (Updegraff et al., 2011; Yardley et al., 2011), subjective norms (Updegraff et al., 2011; Yardley et al., 2011), perceived behavioural control (Liu et al., 2019; Yardley et al., 2011), perceived risk of infection/disease severity (Liu et al., 2019; Updegraff et al., 2011; Yardley et al., 2011), action control (Reyes Fernández et al., 2015), coping planning (Reyes Fernández et al., 2015), knowledge (Liu et al., 2019; Or et al., 2020), or skills (Or et al., 2020).

### Acceptability

Fourteen studies reported on the acceptability of interventions. Indicators assessed included mask comfort (Aiello et al., 2012; Barasheed et al., 2014; Canini et al., 2010; MacIntyre et al., 2009; Suess et al., 2011, 2012), skin problems/irritation (Azor‐Martinez et al., 2016, 2018; Little et al., 2015; Nandrup‐Bus, 2009; Sandora et al., 2005), adverse events (Cowling et al., 2008), liking/positive impressions (Arbogast et al., 2016), and ease of understanding (Updegraff et al., 2011), with a small number of participants experiencing discomfort or irritation in the majority of studies that reported these outcomes (see Table 4).

**Table 4:** APEASE criteria, reach, and engagement

Lead author (year)	Acceptability	Practicability	Affordability	Spill‐over effects	Equity	Reach	Engagement	How primary outcome was assessed	Effectiveness
(1) Aiello (2010)	Not reported	Not reported	Not reported	Not reported	Not reported	7/15 residence halls were included in the study	Not reported	Self‐report	On average, the mask only group wore masks 3.9 hrs per day (*SD* = 3.3) vs. 3.0 hr per day (*SD* = 2.4) in the mask and hand hygiene group (‐). Log mask hours were significantly higher in the mask only group compared with the mask and hand hygiene group at each time point except for week 4 (*p* < .05). The mask only group washed their hands 8.2 times per day (*SD* = 9.0) vs. 6.1 times per day (*SD* = 4.8) in the mask and hand hygiene group and 8.8 times per day (*SD* = 9.3) in the control group (‐). On the log scale, the mask and hand hygiene group washed their hands significantly fewer times per day than the control group from weeks 2 through 4. The mask only group used hand sanitizer 2.3 times per day (*SD* = 3.5) vs. 5.2 times per day (*SD* = 5.1) in the mask and hand hygiene group and 2.0 times per day (*SD* = 3.9) in the control group (+). On the log scale, participants in the mask and hand hygiene group reported a significantly greater use of hand sanitizer compared to the mask only and control groups at each week (*p* < .0001). There were no significant differences between the mask only group and control group in the frequency of hand sanitizer use
(2) Aiello (2012)	On average, participants in the two intervention groups rated mask comfort as 4.7/10 (*SD* = 0.2)	Not reported	Not reported	Not reported	Not reported	5/15 residence halls were included in the study	Not reported	Self‐report and observation of mask wearing by trained staff	Participants in the face mask and hand hygiene group and the face mask only group wore masks on average 5.1 hrs per day (*SD* = 2.2) and 5.0 hrs per day (*SD* = 2.2), respectively (*p* > .05) (/). The face mask and hand hygiene group and the face mask only group reported an average use of hand sanitizer 4.5 times per day (*SD* = 4.1) and 1.3 times per day (*SD* = 1.8), respectively (*p* < .05). This compares with 1.5 times per day (*SD* = 2.3) in the control group (+). The face mask and hand hygiene group and face mask only group washed their hands 5.2 (*SD* = 3.3) and 5.5 (*SD* = 3.3) times per day, respectively. The control group washed their hands an average of 5.8 times (*SD* = 5.0) per day (*p* > .05) (/)
(3) Apisarnthanarak (2009)	Not reported	Not reported	Not reported	Not reported	Not reported	Not reported	Not reported	Observation of hand hygiene and cough etiquette with a Web camera 1 hr, twice weekly	The observed rate of hand hygiene compliance increased from 21% (50/240) in period 1 to 71% (170/240) in period 2 (*p* < .001) and to 69% (166/240) in period 3 (*p* < .001) (+)
(4) Arbogast (2016)	Employees in the intervention group were significantly more likely than those in the control group to have a positive impression of the programme because of the presence of alcohol‐based hand sanitizers in the workplace (80 vs. 69%, *p* < .001). 88% of employees in the intervention group reported liking the products provided	Not reported	Not reported	Not reported	Not reported	1,386/1,609 employees agreed to participate	Not reported	Self‐report	Self‐reported hand washing frequency improved significantly over time in the intervention group (*p* < .05), as did self‐reported hand sanitizer use for every activity assessed, including before eating, after sneezing, coughing, handling money, using the restroom, returning to their desk, and interacting with others who may be sick (*p *< .05) (+)
(5) Azman (2013)	Not reported	Not reported	Not reported	Not reported	Age and ethnicity were significant risk factors for self‐reported influenza‐like illness (*p* < .05)	Not reported	Not reported	Self‐report	Not reported
(6) Azor‐Martinez (2016)	One child showed worsening of existing atopic dermatitis due to hand sanitizer gel use and was excluded from the study	Not reported	Not reported	Not reported	Predictors of a lower rate of absenteeism due to respiratory illness included older age, higher parental income, and correct handwashing technique (*p* < .05)	1,616/1,640 children were randomized	Not reported	Self‐report	Not reported
(7) Azor‐Martinez (2018)	One child showed worsening of existing localized atopic dermatitis due to hand sanitizer gel use and was excluded from the study	Not reported	Not reported	Not reported	Not reported	25/52 daycare centres contacted were randomized	Not reported	Self‐report	Not reported
(8) Barasheed (2014)	The most commonly reported reason for not wearing face masks was discomfort (15% of participants)	Not reported	Not reported	Not reported	Not reported	164/4200 pilgrims took part	Not reported	Self‐report	Compliance with face mask use was 76% (56/75) in the intervention group and 12% (11/89) in the control group (*p* < .001) (+)
(9) Bundgaard (2020)	Not reported	Not reported	The authors note that costs and availability may reduce the efficacy of face masks to prevent SARS‐CoV‐2 infection	Not reported	Not reported	17,258/6304 of those responding to recruitment adverts were randomized	Not reported	Self‐report	46% of participants in the intervention arm wore the mask as recommended, 47% predominantly as recommended, and 7% not as recommended. Participants used an average of 1.7 masks per weekday and 1.3 per weekend day (@)
(10) Canini (2010)	75% of participants in the intervention arm reported discomfort with mask use. The three main causes of discomfort were warmth (45%), respiratory difficulties (33%), and humidity (33%). Children wearing child size face masks reported feeling pain more frequently than those wearing adult face masks (*p* = .036)	Not reported	Not reported	Not reported	Not reported	95/105 randomized households completed the study		Self‐report	Index patients in intervention households reported wearing a total of 11.0 (*SD* = 7.2) masks during 4.0 (*SD* = 1.6) days with an average use of 2.5 (*SD* = 1.3) masks per day and a duration of use of 3.7 (*SD* = 2.7) hours per day (@)
(11) Chan (2007)	Not reported	Not reported	Not reported	Not reported	Not reported	182/295 registered members were successfully contacted; 122/182 took part	Not reported	Self‐report	Significant improvements were observed with regards to washing hands after sneezing/coughing (*M*_before_ = 1.26, *SD* = 0.44; *M*_after_ = 1.65, *SD* = 0.89), washing hands with liquid soap (*M*_before_ = 1.14, *SD* = 0.35; *M*_after_ = 1.53, *SD* = 0.82), and wearing masks in public (*M*_before_ = 1.31, *SD* = 0.46; *M*_after_ = 2.15, *SD* = 1.12) (all *p* < .0001) (+)
(12) Cowling (2008)	There were no reported adverse events requiring medical attention	Not reported	Not reported	Not reported	Not reported	198/944 index patients were randomized	Not reported	Self‐report	In the face mask group, 45% (10/22) of index cases reported wearing a mask often or always, compared with 30% (22/74) and 28% (9/32) in the control and hand hygiene groups, respectively (+). In the face mask group, 21% (14/65) of household contacts reported wearing a mask often or always, compared with 1% (2/213) and 4% (4/92) in the control and hand hygiene groups, respectively. Index cases in the face mask group used a median of 12 masks (IQR = 6–18) whereas household contacts used a median of six masks (IQR = 1–20). 63% (41%) of index cases (household contacts) in the hand hygiene group reported washing their hands often or always after sneezing, coughing or cleaning their nose, compared with 63% (47%) and 31% (27%) in the face mask and control groups, respectively (+). In the hand hygiene group, households used a median of 56 g (IQR = 27–93) of alcohol from the automatic sanitizer and a median of 88 g (IQR = 63–149) of liquid hand soap over the course of the study
(13) Cowling (2009)	Not reported	Not reported	Not reported	Not reported	Not reported	407/2,750 index patients and their households were randomized	Not reported	Self‐report	The proportion index cases wearing face masks was 15% in the control group (14/91); 31% in the hand hygiene group (26/85); and 49% (41/83) in the face mask and hand hygiene group (+). The proportion household contacts wearing face masks was 7% (20/279) in the control group; 5% (13/257) in the hand hygiene group; and 26% (67/258) in the face mask and hand hygiene group. The median (IQR) use of liquid soap in the hand hygiene group was 77.6 g (42.4–162.6) and 78.9 (35.2–114.2) in the face mask and hand hygiene groups. The median (IQR) hand rub used in the hand hygiene group was 3.2 g (1.1–9.7) in index cases and 1.5 g (0.3–5.3) in contacts. This compared with 1.6 g (0.7–5.1) in index cases and 1.5 g (0.3–3.8) in contacts in the face mask and hand hygiene group (/)
(14) Hübner (2010)	Not reported	Not reported	Not reported	Not reported	Not reported	134/850 participants were randomized	Not reported	Self‐report	The mean hand disinfection frequency was >5 times daily in 19%, 3–5 times daily in 60%, and 1–2 times daily in 21% (@)
(15) Kaewchana (2012)	Not reported	Not reported	Not reported	Not reported	Not reported	Not reported	Not reported	Self‐report	On day 7, the control and intervention groups reported 3.9 (*SD* = 2.4) and 5.7 (*SD* = 3.4) hand washing episodes/day, respectively (*p* < .001) (+). The percentage of participants who used soap increased from 34% (53/158) to 88% (139/158) (*p* < .001)
(16) Koep (2016)	Not reported	Not reported	Not reported	Not reported	Not reported	Not reported	Not reported	Self‐report and objective measure of soap and sanitizer use	No significant differences in hand soap compliance were observed across groups (estimates not reported) (/). Hand sanitizer use increased slightly in the intervention school from 0.04 to 0.06 times per student per day in grades 3 and 4 (*p* > .05)
(17) Larson (2009)	Not reported	Not reported	Not reported	Hand sanitizer use increased but hand washing with soap decreased	Respondents with college degrees had higher knowledge scores than the other groups, adjusting for baseline scores (*p* = .04)	Not reported	Not reported	Self‐report (questionnaire administered by trained researchers) and objective measures of hand sanitizer and mask use	A significantly greater proportion of participants reported using hand sanitizer at the end of the study (282/422) compared with baseline (6/422) (+), but a lower proportion reported using antibacterial soap at the end of the study (105/422) compared with baseline (191/422) (‐)
(18) Larson (2010)	Not reported	Not reported	Not reported	Not reported	Not reported	617/672 households that expressed interest in participation met eligibility criteria; 509/617 completed the initial home visit	Not reported	Self‐report (questionnaire administered by trained researchers) and objective measures of hand sanitizer and mask use	Participants in the hand sanitizer group used a mean of 12.1 ounces/month and those in the hand sanitizer and face mask group used a mean of 11.6 ounces/month (*p* = .36) (/). Half of the households with a case of infection reported using masks within 48 hrs of symptom onset. Those who used masks reported a mean of two masks/day/episode (range: 0–9) (@)
(19) Little (2015)	Minor self‐reported skin irritation increased among those who did not report problems at baseline. Among individuals who had a (skin complaint at baseline, reported skin complaints did not significantly increase over time)	Not reported	Not reported	Not reported	Not reported	20,066/80,4897 who received a mailed invitation were randomized	Not reported	Self‐report	Not reported
(20) Liu (2019)	Not reported	Not reported	Not reported	Not reported	Not reported	12/213 kindergarten clusters received the intervention	Not reported	Self‐report	The average self‐reported compliance with hand hygiene guidelines was significantly greater after the intervention (9.7, IQR = 0.23) compared with baseline (9.4, IQR = 0.47), *p* < .01 (+). Teachers reported significantly more hand washing behaviour compared with baseline after coughing/sneezing, blowing their nose, changing a diaper, contacting bodily fluids and soiled textiles, going to the toilet, wiping the nose of a child, wiping a child’s bottom, and before helping a child with food (*p* < .05) (+)
(21) MacIntyre (2009)	There were no significant differences in difficulties with mask use between the P2 and surgical mask groups, but >50% reported concerns, the main one being that wearing a face mask was uncomfortable. Other concerns were that the child did not want the parent wearing a mask. Some participants mentioned that the mask did not fit well and that it was not practical to wear at mealtime or while asleep	Fit testing for P2 masks was not conducted because this was judged unlikely to be feasible in the general community during a pandemic	Not reported	Not reported	Not reported	145/401 families assessed for eligibility took part	Not reported	Self‐report	On day 1 of mask use, 38% (36/94) of the surgical mask users and 46% (42/92) of the P2 mask users stated that they were wearing the mask ‘most or all of the time’ (*p* = .37) (/). Adherence dropped to 31% (29/94) and 25% (23/92), respectively, by day 5 of mask use
(22) Mott (2007)	Not reported	Not reported	Not reported	Hand sanitizer use increased in leaders in both intervention groups (from 3.0 to 13.4 times/day and from 3.2 to 4.7 times/day, respectively)	Not reported	Not reported	Not reported	Self‐report	Post‐intervention, there was a decrease in the daily frequency of hand washing in the SI group (from 4.4 to 2.6 times/day) (‐) and no change in the PI group (from 4.9 to 5.0 times/day). Hand sanitizer use increased in both intervention groups (from 3.7 to 10.4 times/day and from 4.0 to 6.0 times/day, respectively) (+)
(23) Nandrup‐Bus (2020)	Three children withdrew from the intervention arm due to skin problems	Not reported	Not reported	Not reported	Not reported	Not reported	Not reported	Objective measure of soap use	School records showed a usual monthly consumption of 2–2.5 litres of liquid soap at the intervention school. During the intervention period, consumption increased to 16 L of liquid soap (but accurate monthly measurement was not possible as soap was continuously replenished). No reliable measurement of soap consumption in the control school was available (@)
(24) Or (2020)	Not reported	Not reported	Not reported	Not reported	Not reported	Not reported	Not reported	Self‐report and observation of hand washing with fluorescent stain gel and photographs	After the programme, the percentages of properly washed areas on both hands increased significantly, in particular the wrists (from 0.5 to 82%), *p* < .001 (+)
(25) Ram (2015)	Not reported	Not reported	Not reported	Not reported	Not reported	377/766 eligible index cases took part	Not reported	The intervention staff weighed the soap each day and replaced it if the bar weighed <20 g	A median per capita soap consumption of 2.3 g (IQR = 1.7–3.2) was observed in the first 12 days of the programme (@)
(26) Reyes Fernández (2015)	Not reported	Not reported	Not reported	Not reported	Not reported	242/440 students completed the study	Not reported	Self‐report	Self‐reported frequency of hand sanitizer use per day, measured on a 5‐point Likert scale, increased in both control (from *M* = 1.5, *SD* = 0.9 to *M* = 1.7, *SD* = 1.3) and intervention groups (from *M* = 1.8, *SD* = 1.3 to *M* = 2.1, *SD* = 1.4), *p* = .09 (/)
(27) Roberts (2000)	Not reported	Not reported	Not reported	Not reported	Not reported	23/26 eligible childcare centres took part	Not reported	Observation by trained researchers	Hand washing compliance in children was divided into three groups, corresponding to intervention centres with a score of low (53–69%; four centres), moderate (70–79%; four centres), and high (>80%; three centres) compliance (@)
(28) Sandora (2005)	Forty‐five families reported 112 adverse events related to hand sanitizer use. Seventy‐one (63%) of the adverse events were in relation to ‘dry skin’, and 20 (18%) were related to ‘irritation’. Other reported adverse events included ‘stinging’, ‘smells bad’, ‘dislike it’, ‘allergic reaction’, and ‘too slippery’	Not reported	Not reported	Not reported	Not reported	292/647 families assessed for eligibility took part	Not reported	Self‐report	Primary caregivers reported using the hand sanitizer with a median frequency of 5.2 times/day (@)
(29) Savolainen‐Kopra (2012)	Not reported	Not reported	Not reported	Not reported	Not reported	683/1,270 employees considered for eligibility took part	Not reported	Assessment by study nurses	The average amount of soap or disinfectant use per participant was 6.1 and 6.9 g in the active groups (@)
(30) Simmerman (2011)	Not reported	Not reported	Not reported	Not reported	Not reported	465/20,537 paediatric outpatients with influenza‐like illness took part	Not reported	Self‐report	Participants in the hand washing group reported 4.7 washing episodes/day, compared with 4.9 times/day in the hand washing plus face mask arm and 3.9 times/day in controls (*p* = .001) (+). Participants in the face mask arm used an average of 12 masks per person, per week (median = 11, IQR = 7–16) and reported wearing face masks a median of 211 min/day (IQR = 17–317) (@). Parents wore their masks for a median of 153 (IQR = 40–411) minutes per day, which was greater than other relations (median = 59; IQR = 9–266), index cases themselves (median = 35; IQR = 4–197), or their siblings (median = 17; IQR = 6–107)
(31) Stebbins (2010)	Not reported	Not reported	Not reported	Not reported	Not reported	151/167 enrolled teachers took part	Not reported	Self‐report	The proportion of students washing their hands more than three times per day in the post‐flu season was significantly greater in the intervention arm (*M* = 3.7) compared with the control arm (*M* = 3.4), *p* = .04 (+)
(32) Stedman‐Smith (2015)	Not reported	Not reported	Not reported	Not reported	Not reported	324/1,708 enrolled office workers took part	Not reported	Self‐report	In both intervention and control groups, >80% of employees reported hand sanitizer use at least 25% of the time (*p* > .05) (/)
(33) Suess (2011)	The majority (60%) of participants did not report any problems when wearing face masks. Of those who reported having removed their masks in transmission‐prone situations, ‘feeling hot’ was the main reason. Other problems mentioned less frequently were pain when wearing the mask and shortness of breath. The majority of adult household contacts in the control and intervention groups perceived wearing face masks as well as intensified hand hygiene as an effective means of preventing transmission of influenza	Not reported	Not reported	Not reported	Not reported	Not reported	Not reported	Self‐report	81% of index cases and 71% of household members from the combined active groups wore a mask ‘always’ or ‘most of the time’ when in the same room with either a healthy or an infected person (@). Participants (index cases and contacts combined) in the mask plus hygiene group washed/disinfected their hands significantly more frequently (51/56) compared with those in the mask and control groups combined (59/89) (*p* = .007) (+)
(34) Suess (2012)	The majority of participants (62%) did not report any problems with mask wearing. This proportion was significantly higher in adults (71%) than children (50%) (*p* = .005). The main problem stated by participants (adults as well as children) was ‘heat/humidity’, followed by ‘pain’ and ‘shortness of breath’ when wearing a face mask	Not reported	Not reported	Not reported	Not reported	Not reported	Not reported	The amount of remaining intervention materials was assessed at the end of the study period	Participants in the mask group used a median of 12.9 (IQR = 9.5–16) face masks per individual. Participants in the mask plus hygiene group used a median of 12.6 (IQR = 7.8–14) face masks (@). The number of hand disinfections per day was 7.4 and 4.1 in index cases in the 2009/10 and 2010/11 cohorts, respectively. The number of hand disinfections per day was 8.8 and 7.5 in household contacts in the 2009/10 and 2010/11 cohorts, respectively
(35) Updegraff (2011)	There were no significant differences between the four signs in how easy they were to understand. The loss‐framed and perceived susceptibility signs elicited significantly more negative affect than the gain‐framed and norms signs (*p* < .01)	Not reported	Not reported	Not reported	Not reported	Not reported	Not reported	Grams of sanitizer used per day, measured with a digital scale	All signs resulted in significantly greater hand sanitizer use compared with the no sign condition (+), but they were not equally effective. Dispensers with the gain‐framed signs had the greatest hand sanitizer use, with 66.4% more use than dispensers with no signs (*p* < .001). Loss‐framed signs were associated with a 58.4% increase in use compared with no sign (*p* < .001). The social norms signs (44.3% increase) and the perceived susceptibility signs (40.6% increase) were associated with somewhat lower increases in usage compared with the gain‐framed and loss‐framed signs, but both led to significantly more use than no sign (both *p* < .01)
(36) White (2003)	Not reported	Not reported	Not reported	Not reported	Not reported	Not reported	Not reported	Self‐report	Over the course of the study, the product group washed their hands 10.4% more frequently than the control group (0.5 times/hr vs. 0.4 times/hr; *p* < .05) (+). The frequency of hand sanitizer use was also significantly greater in the product group (0.3 times/hr vs. 0.0 times/hr; *p* < .05)
(37) Yardley (2011)	Not reported	Not reported	Not reported	Not reported	Moderator analyses indicated that the intervention was similarly effective for those from higher and lower socio‐economic status groups	517/8,150 of those invited participated	Of the 324 participants who were randomly assigned to the intervention, 251 (77.5%) progressed to the second session, 219 (67.6%) completed three sessions, and 188 (58.0%) completed all four sessions. The free hand gel was collected by 170/324 (52.5%) eligible participants	Self‐report	Significant differences between groups in hand washing frequency, measured on a 5‐point scale, were observed at 4 weeks (*M* = 4.4, *SD* = 0.9 vs. *M* = 4.0, *SD* = 0.9; Cohen’s *d* = .42) and at 12 weeks (*M* = 4.1, *SD* = 1.1 vs. *M* = 4.5, *SD* = 0.8; Cohen’s *d* = .34), *p* < .001 (+)
(38) Zomer (2016)	Not reported	Not reported	Due to budget restrictions, hand hygiene products were only provided for two groups within each daycare centre, even if the centre had more than two groups	No significant effect of the intervention was found on supervising children’s hand hygiene (36 vs. 32%)	Not reported	71/122 daycare centres participated	Of 274 caregivers, 21% (54/261) attended none of the training sessions, 25% (66/261) attended one training session, 29% (75/261) attended two training sessions and 25% (66/261) attended all three sessions	Direct observation by trained researchers	Hand hygiene compliance in intervention daycare centres was 66 vs. 43% in control centres (OR = 6.33, 95% CI = 3.71–10.80) (+)
(39) Öncü (2019)	Not reported	Not reported	Not reported	Not reported	Not reported	96/552 children	Not reported	Photographs examined by researchers not directly involved in the study	In the most intensive intervention group, post‐intervention hand washing effectiveness scores increased significantly across all regions of both hands (*p* < .05) (+)

Note

+ = positive effect; − = negative effect; / = no difference; @ = indeterminate; and NA = not applicable.

### Practicability

One study (MacIntyre et al., 2009) considered the practicability of scaling up mask fit testing outside the study setting and decided against including routine fit testing as part of the intervention (see Table 4).

### Effectiveness

Studies relied on self‐report (25/39; 64%), direct observation (7/39; 18%), a combination of self‐report and direct observation (5/39; 13%), or photographs/video (2/39; 5%) to examine intervention effectiveness (see Table 4). Outcome variables were heterogeneous across studies (e.g., the frequency or amount of hand sanitizer/soap use per day, the rate of compliance with hand hygiene, or the rate of compliance with face mask use).

#### Hand hygiene

**Figure 4:**
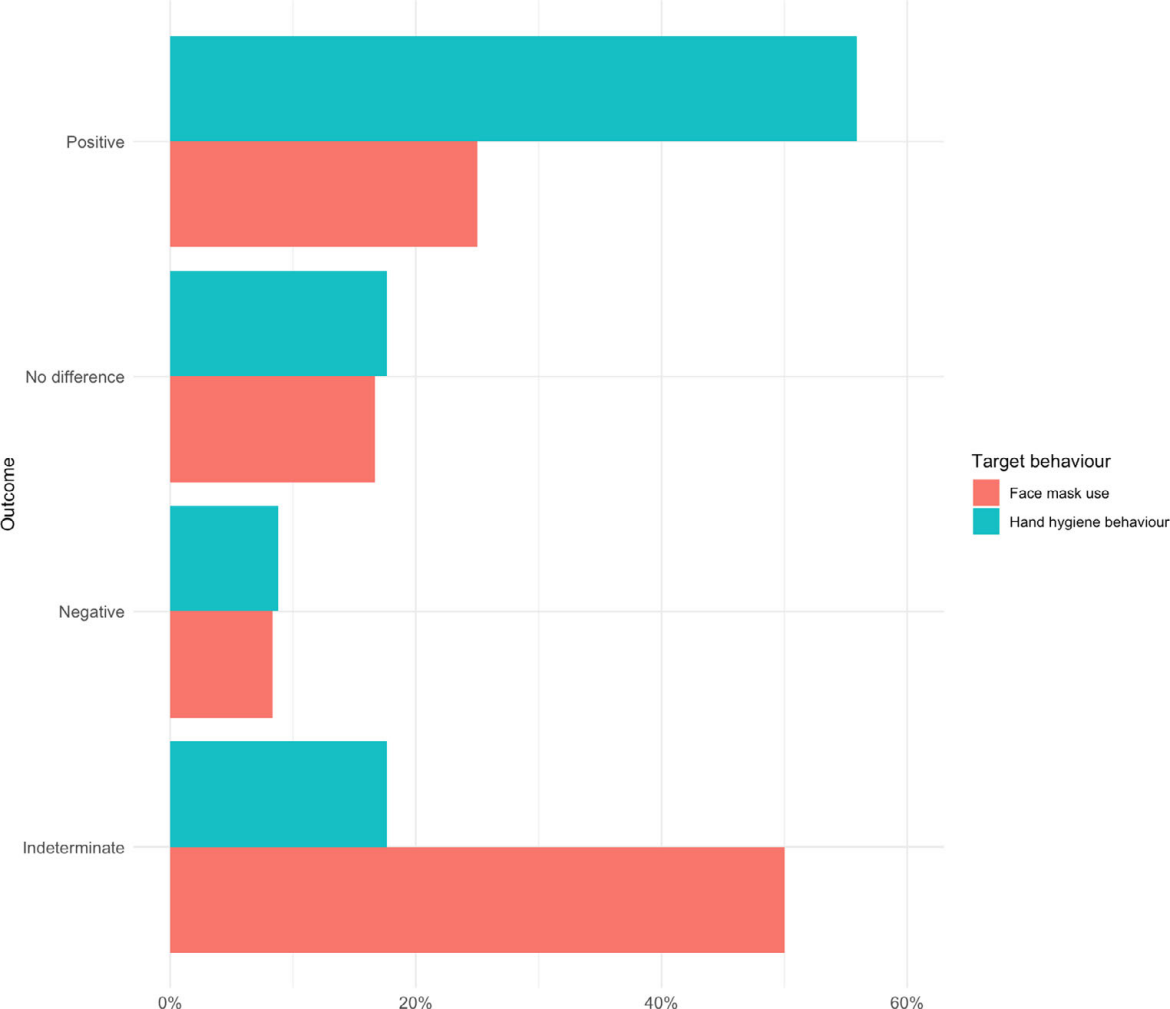
Proportions of reported effects (i.e., positive, negative, no difference, or indeterminate) of interventions targeting hand hygiene behaviours and face mask use.

Overall, the 30 studies pertaining to hand hygiene behaviours (including hand washing and/or hand sanitizer/soap use) had positive results, with 19 studies reporting positive effects (Aiello et al., 2010, 2012; Apisarnthanarak et al., 2009; Arbogast et al., 2016; Chan et al., 2007; Cowling et al., 2008; Kaewchana et al., 2012; Larson et al., 2009; Liu et al., 2019; Mott et al., 2007; Öncü et al., 2019; Or et al., 2020; Simmerman et al., 2011; Stebbins et al., 2010; Suess et al., 2011; Updegraff et al., 2011; White et al., 2003; Yardley et al., 2011; Zomer et al., 2016), three studies reporting negative effects (Aiello et al., 2010; Larson et al., 2009; Mott et al., 2007), six studies reporting no difference (Aiello et al., 2012;; Cowling et al., 2009; Koep et al., 2016; Larson et al., 2010; Reyes Fernández et al., 2015; Stedman‐Smith et al., 2015), and six studies with indeterminate results (Hübner et al., 2010; Nandrup‐Bus, 2009; Ram et al., 2015; Roberts et al., 2000; Sandora et al., 2005; Savolainen‐Kopra et al., 2012) (see Figure 4). It should be noted that some studies reported more than one hand hygiene outcome (e.g., hand washing and hand sanitizer use).

**Figure 5:**
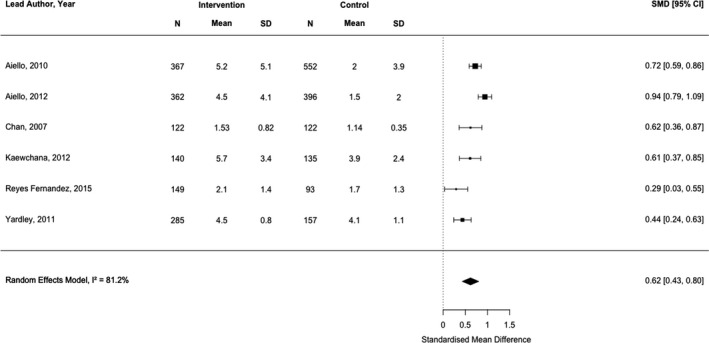
Forest plot for the standardized mean difference (*d*) in the frequency of hand hygiene behaviour in intervention and control or pre‐ and post‐study comparisons. The comparison in Chan (Chan et al., 2007) pertains to a pre‐ and post‐study comparison; the remaining studies were two‐ or three‐arm RCTs.

A random‐effects meta‐analysis (*k* = 6) found a medium, positive effect of interventions on the average frequency of hand hygiene behaviour, *d* = .62, 95% CI = 0.43–0.80, *p* < .001 (see Figure 5). However, between‐study heterogeneity was high (*I* ^2^ = 81.2%).

#### Face mask use

Overall, the 12 studies pertaining to face mask use reported mixed results, with three studies reporting positive effects (Barasheed et al., 2014; Cowling et al., 2008, 2009), two studies reporting no difference (Aiello et al., 2012; MacIntyre et al., 2009), one study reporting negative effects (Aiello et al., 2010), and six studies with indeterminate results (Bundgaard et al., 2020; Canini et al., 2010; Larson et al., 2010; Simmerman et al., 2011; T Suess et al., 2011; Suess et al., 2012) (see Figure 4).

**Figure 6:**
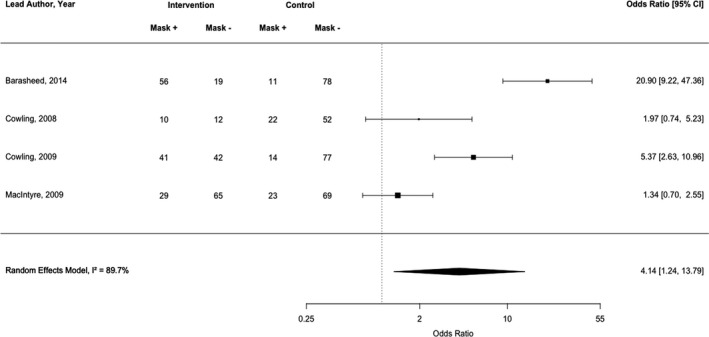
Forest plot for the odds of compliance with face mask use in intervention compared with control arms. The comparison in MacIntyre (MacIntyre et al., 2009) pertains to the surgical mask (intervention) vs. the P2 mask arm (control) at the longest point of follow‐up.

A random‐effects meta‐analysis (*k* = 4) found a large, positive effect of interventions on the odds of compliance with face mask use, OR = 4.14, 95% CI = 1.24–13.79, *p* < .001 (see Figure 6). However, between‐study heterogeneity was high (*I* ^2^ = 89.67%) and the confidence interval for the pooled effect was wide.

### Affordability

Two studies considered the affordability of interventions, with one study discussing the cost of face masks (Bundgaard et al., 2020), which may act as a barrier for wider roll‐out, and a second study (Zomer et al., 2016) reporting that although they wanted to provide hand hygiene products to all daycare centre groups, they could only afford to do so for a maximum of two groups per centre due to budget restrictions, thus indicating that the selected intervention was not affordable at scale (see Table 4).

### Spill‐over effects

Three studies reported on secondary behaviour change (i.e., positive or negative spill‐over to other behaviours), with one study (Zomer et al., 2016) assessing teachers’ supervision of children’s hand washing (in addition to their own hand washing), a second (Mott et al., 2007) assessing the impact of the intervention on military leaders’ hand sanitizer use (in addition to trainees’), and a third (Larson et al., 2009) examining multiple hand hygiene behaviours in the same group of participants. The first study reported no change, the second a significant increase in hand sanitizer use, and the third a negative impact on hand washing with soap (see Table 4).

### Equity

Four studies reported on the equity of interventions, with one study (Yardley et al., 2011) reporting that the intervention was equally effective for participants from high and low socio‐economic status groups and three studies (Azman et al., 2013; Azor‐Martinez et al., 2016; Larson et al., 2009) reporting differential intervention effectiveness by educational attainment, parental income, or ethnicity, with better outcomes reported in those with high educational attainment, high parental income, and from a Black ethnic background (see Table 4).

### Quality of included studies

One study received an overall rating of ‘low risk of bias’, with 16 studies rated as ‘some concern’, 18 as ‘high risk of bias’, and for four studies, an overall rating could not be applied (see Table 5).

**Table 5:** Quality appraisal

Lead author (year)	(1) Bias arising from the randomization process	(2) Bias due to deviations from the intended interventions	(3) Bias due to missing outcome data	(4) Bias in measurement of the outcome	(5) Bias in selection of the reported result	Overall rating
(1) Aiello (2010)	Some concerns	Low risk of bias	Some concerns	Low risk of bias	Low risk of bias	Some concerns
(2) Aiello (2012)	Some concerns	Low risk of bias	Some concerns	Some concerns	Low risk of bias	Some concerns
(3) Apisarnthanarak (2009)	NA	NA	NA	NA	NA	NA
(4) Arbogast (2016)	Some concerns	Some concerns	Low risk of bias	Low risk of bias	Some concerns	Some concerns
(5) Azman (2013)	Some concerns	Some concerns	Low risk of bias	Some concerns	Some concerns	Some concerns
(6) Azor‐Martinez (2016)	Some concerns	Some concerns	Some concerns	Some concerns	Low risk of bias	High risk of bias
(7) Azor‐Martinez (2018)	Some concerns	Some concerns	Low risk of bias	Low risk of bias	Low risk of bias	Some concerns
(8) Barasheed (2014)	Some concerns	Some concerns	Some concerns	Some concerns	Some concerns	High risk of bias
(9) Bundgaard (2020)	Some concerns	Some concerns	Some concerns	Some concerns	Low risk of bias	High risk of bias
(10) Canini (2010)	Some concerns	Some concerns	Low risk of bias	Low risk of bias	Low risk of bias	Some concerns
(11) Chan (2007)	NA	Some concerns	Some concerns	Some concerns	Some concerns	High risk of bias
(12) Cowling (2008)	Some concerns	Some concerns	Some concerns	Low risk of bias	Low risk of bias	Some concerns
(13) Cowling (2009)	Some concerns	Some concerns	Some concerns	Low risk of bias	Low risk of bias	Some concerns
(14) Hübner (2010)	Some concerns	Some concerns	Low risk of bias	Some concerns	Some concerns	High risk of bias
(15) Kaewchana (2012)	Some concerns	Some concerns	Some concerns	Some concerns	Some concerns	High risk of bias
(16) Koep (2016)	Some concerns	Some concerns	Some concerns	Some concerns	Some concerns	High risk of bias
(17) Larson (2009)	NA	Some concerns	Some concerns	Some concerns	Some concerns	High risk of bias
(18) Larson (2010)	Some concerns	Some concerns	Low risk of bias	Some concerns	Some concerns	High risk of bias
(19) Little (2015)	Some concerns	Some concerns	Some concerns	Low risk of bias	Some concerns	High risk of bias
(20) Liu (2019)	NA	NA	NA	NA	NA	NA
(21) MacIntyre (2009)	Some concerns	Some concerns	Low risk of bias	Some concerns	Low risk of bias	Some concerns
(22) Mott (2007)	NA	NA	NA	NA	NA	NA
(23) Nandrup‐Bus (2020)	Some concerns	Some concerns	Some concerns	Some concerns	Low risk of bias	High risk of bias
(24) Or (2020)	NA	NA	NA	NA	NA	NA
(25) Ram (2015)	Some concerns	Some concerns	Low risk of bias	Low risk of bias	Low risk of bias	Some concerns
(26) Reyes Fernández (2015)	Some concerns	Some concerns	Some concerns	Some concerns	Low risk of bias	High risk of bias
(27) Roberts (2000)	Some concerns	Some concerns	Some concerns	Low risk of bias	Some concerns	High risk of bias
(28) Sandora (2005)	Low risk of bias	Some concerns	Some concerns	Some concerns	Some concerns	High risk of bias
(29) Savolainen‐Kopra (2012)	Some concerns	Some concerns	Some concerns	Some concerns	Low risk of bias	High risk of bias
(30) Simmerman (2011)	Low risk of bias	Low risk of bias	Some concerns	Some concerns	Low risk of bias	Some concerns
(31) Stebbins (2010)	Some concerns	Some concerns	Low risk of bias	Some concerns	Some concerns	High risk of bias
(32) Stedman‐Smith (2015)	Low risk of bias	Low risk of bias	Some concerns	Some concerns	Some concerns	Some concerns
(33) Suess (2011)	Low risk of bias	Low risk of bias	Some concerns	Some concerns	Some concerns	Some concerns
(34) Suess (2012)	Low risk of bias	Some concerns	Some concerns	Some concerns	Some concerns	High risk of bias
(35) Updegraff (2011)	Low risk of bias	Low risk of bias	Low risk of bias	Low risk of bias	Low risk of bias	Low risk of bias
(36) White (2003)	High risk of bias	Low risk of bias	Some concerns	Some concerns	Some concerns	High risk of bias
(37) Yardley (2011)	Low risk of bias	Low risk of bias	Some concerns	Some concerns	Low risk of bias	Some concerns
(38) Zomer (2016)	Low risk of bias	Some concerns	Low risk of bias	Some concerns	Low risk of bias	Some concerns
(39) Öncü (2019)	Some concerns	Some concerns	Low risk of bias	Low risk of bias	Some concerns	Some concerns

Note

NA = not applicable.

## Discussion

This rapid review of interventions to increase personal protective behaviours to limit the spread of respiratory viruses identified 39 studies conducted across 15 countries. The majority of interventions targeted hand hygiene and/or face mask use, with one intervention targeting the catching of droplets in tissues in addition to hand hygiene. None of the identified interventions focused on avoiding touching the T‐Zone, disinfecting surfaces or maintaining physical distancing. Interventions were typically delivered in participants’ own homes or in nurseries/schools, targeting children/adult household members or pre‐ or school children/teachers. Two‐ or three‐arm study designs with passive comparators were typically used. The overall quality of included studies was low, with only one study rated as ‘low risk of bias’. The majority of interventions had a face‐to‐face component and delivered a median of three BCTs; the most frequent were ’Adding objects to the environment’, ‘Instruction on how to perform the behaviour’ and ‘Information about health consequences’. Where investigated, interventions were considered acceptable by participants, with a minority reporting issues with mask wear discomfort or skin irritation from hand hygiene products. Few studies reported the practicability, affordability, spill‐over effects or equity of interventions. In a narrative synthesis, interventions targeting hand hygiene behaviour were found to have positive effects and those targeting face mask use had a mixture of positive and negative effects. Random‐effects meta‐analyses of a small number of studies found positive effects of interventions targeting hand hygiene behaviour and face mask use. However, between‐study heterogeneity was high and the confidence interval for the pooled effect of interventions targeting face mask use was wide, partly due to the small number of studies included in the comparison.

### Strengths and limitations

This review was conducted rapidly (July–December 2020) with input on the research questions and review scope from public health and behavioural science experts and lay members as part of a written stakeholder consultation. However, the pragmatic nature of this review, conducted during an ongoing pandemic, also means that it has several important limitations. First, given the expected large number of hand hygiene studies related to gastrointestinal infections, we limited the review to studies explicitly studying behaviour change in relation to respiratory viruses. However, data from interventions targeting personal protective behaviours to prevent gastrointestinal illness are likely to add to our understanding of the acceptability, effectiveness, and equity of hand hygiene interventions. Second, evidence indicates that the relative importance of different personal protective behaviours may depend on properties of the specific respiratory virus and context (e.g., fomite transmission may be more pronounced for respiratory syncytial virus compared with coronaviruses) (Boone & Gerba, 2007). However, at the time of planning this rapid evidence review, little was known about SARS‐CoV‐2. We therefore opted for a broad scope and included interventions targeting personal protective behaviours to limit the spread of any respiratory viral infection. It was also not possible to group the results based on the specific viral infections studied as the majority of studies targeted multiple (as opposed to single) respiratory viral infections and there was little variability in the viral infections targeted. In addition, as the majority of studies targeted a host of different viruses within their interventions, this further limits the conclusions that can be drawn: The perceived susceptibility to different viruses likely differs between, for example, age groups (Rosenstock, 1974), and tailored intervention strategies may therefore be needed for younger (vs. older) adults. However, the current review was unable to address such nuanced questions due to the limited design and reporting of extant studies and the need to synthesize evidence quickly during an ongoing pandemic. Third, our electronic search was restricted to two databases, which may have limited the results, and data extraction was performed by a single reviewer, with a proportion verified by a second reviewer. Fourth, although most of identified studies were two‐ or three‐arm RCTs, they were typically designed to study rates of respiratory infection as their primary outcome, with behaviour change outcomes less clearly reported. This hindered quantitative synthesis, with only a small number of included studies contributing to meta‐analyses. Future studies specifically designed to examine the effectiveness of interventions on behavioural outcomes are needed. Fifth, in line with guidelines (Wood et al., 2015), we only coded BCTs when there was clear evidence of their presence; interventions may have included additional BCTs not documented in this review. Sixth, as this was a rapid review with limited resources, we limited our analyses of intervention content to information presented in the published papers and/or available supplementary materials, and no attempts were made to contact study authors for access to detailed intervention descriptions. Seventh, as most interventions targeting multiple behaviours (e.g., hand hygiene and face mask use) did not clearly distinguish BCTs that targeted one (but not the other) behaviour, and none of the outcome evaluations considered potential behavioural dependencies (or statistical interactions), it was not possible to consider the extent of BCT overlap and/or behavioural interactions in the present review. Finally, due to the small number of studies available for meta‐analysis, we were unable to group studies by, for example, population type, study setting, type of virus, type of outcome assessment, etc., which would have further improved our understanding of intervention effectiveness.

### Implications for policy and practice

Although we caution against drawing firm conclusions due to the low quality of the evidence, positive effects of interventions targeting hand hygiene behaviour and face mask use were observed, with the majority of interventions providing free hand hygiene products and/or face masks to participants in addition to instructions on how to perform the behaviour and information about health consequences. As far as is practicably feasible, authorities should aim to provide free products to staff, clients, and visitors during respiratory viral epidemics. The limited range of BCTs detected in published intervention descriptions may suggest a missed opportunity for harnessing techniques indicated by relevant behaviour change theory and evidence. We encourage policymakers and health care practitioners to work collaboratively with behavioural scientists to incorporate techniques that theory or evidence predicts are effective for enabling personal protective behaviours (Warren‐Gash et al., 2012), such as techniques targeting motivational or self‐regulatory processes. For example, while hand hygiene is a well‐established, often private and widely accepted protective behaviour that most people have long experience with, face mask wearing is a relatively new (at least in some countries), public behaviour, where there is more debate and uncertainties about the impact of the behaviour among the public and scientists (Cowling, Zhou, Ip, Leung, & Aiello, 2010). It is therefore important to involve behavioural scientists in the development of any new interventions targeting personal protective behaviours in the context of respiratory viral infections to help map out potential influences (e.g., social, self‐regulatory) that may help or hinder the target behaviour, acknowledging that different interventions are likely needed for different behaviours.

### Avenues for future research

Findings highlight the need for evaluations of interventions to support people to avoid touching the T‐Zone, disinfect surfaces, maintain physical distancing, and ensure efficient ventilation. Ventilation is increasingly seen as an important personal protective behaviour but was missed from the present review as it was planned during an earlier epidemic phase when the emphasis was on viral transmission via droplets rather than aerosols (Anderson, Turnham, Griffin, & Clarke, 2020; Morawska & Milton, 2020). In addition, we need studies designed to detect effects on behavioural outcomes and data on the affordability and equity of interventions to increase personal protective behaviours, particularly in low‐ and middle‐income countries. Although the provision of hand hygiene products and face masks may offset costs related to primary and secondary care or work absenteeism for those with severe respiratory viral illness, the provision of free products at scale may be prohibitively costly. Future research involving health and social care economists should evaluate the cost‐effectiveness of different types of interventions to enable personal protective behaviours, including those targeting motivational and self‐regulatory processes. We also need further evidence from studies evaluating interventions to improve adherence to face mask use, with unclear results observed at present. Finally, due to the small number of studies with data suitable for meta‐analysis, we did not conduct moderator analyses to examine whether, for example, particular BCTs, broader content categories, or the unit of randomization (e.g., individual vs. cluster) were related to intervention effectiveness; this would be important to examine in future meta‐analyses with larger sample sizes. We did not consider here the use of, for example, the Theoretical Domains Framework (Cane, O’Connor, & Michie, 2012) when coding the mechanisms of action of interventions; this may be useful to consider in future empirical studies and evidence reviews. Finally, although evidence generation during ongoing pandemics is challenging (with a need to balance a pragmatic approach and limited resources with scientific rigour), drawing primarily on studies conducted outside the pandemic setting to inform what behavioural interventions to implement is suboptimal. We therefore recommend that experimental studies of behavioural interventions are prioritized during future respiratory viral pandemics.

### Conclusions

This rapid review identified 39 studies across 15 countries with interventions targeting hand hygiene and/or face mask use. Positive effects of interventions targeting hand hygiene were observed, with unclear results for interventions targeting face mask use. There was a lack of evidence for interventions targeting most behaviours of interest within this review.

## Funding

OP receives salary support from Cancer Research UK (C1417/A22962).

## Conflicts of interest

All authors declare no conflict of interest.

## Author contributions

Olga Perski (Conceptualization; Data curation; Formal analysis; Methodology; Visualization; Writing – original draft; Writing – review & editing) Dorothy Szinay (Data curation; Writing – review & editing) Elizabeth Corker (Conceptualization; Data curation; Methodology; Writing – review & editing) Lion Shahab (Conceptualization; Formal analysis; Methodology; Writing – review & editing) Robert West (Conceptualization; Methodology; Writing – review & editing) Susan Michie (Conceptualization; Methodology; Writing – review & editing).

## Supporting information


**Appendix S1**. Search strategy.

## Data Availability

The extracted data that support the findings of this rapid review are available on request from the corresponding author, OP.
