# Supplementary material for: Interventions to increase personal protective behaviours to limit the spread of respiratory viruses: A rapid evidence review and meta‐analysis
Source: Br J Health Psychol. 2021 Jun 26;27(1):215–64. doi: 10.1111/bjhp.12542 (PMC12086758; doi:10.1111/bjhp.12542)
Supplement: Supplementary file 1 — Appendix S1. Search strategy. [file BJHP-27-215-s001.docx]

**Supplementary file 1**

*Search strategy*

1) Ovid MEDLINE

1. hand hygiene.mp. or Hand Disinfection/ or Infection Control/ or Hand Hygiene/
2. hand washing.mp.
3. face mask.mp.
4. face touching.mp.
5. physical distancing.mp.
6. social distancing.mp.
7. Disinfection/
8. intervention.mp.
9. 1 or 2 or 3 or 4 or 5 or 6 or 7
10. 8 and 9

2) Scopus

TITLE ( "hand hygiene"  OR  "hand washing"  OR  "hand disinfection"  OR  "hand saniti$er"  OR  "infection control"  OR  "mask wearing"  OR  "face mask"  OR  "mask"  OR  "facemasks"  OR  "face coverings"  OR  “personal protective equipment” OR "face touching"  OR  "disinfection"  OR  "physical distancing"  OR  "social distancing"  OR  "self-inoculation" OR  "hand-to-face contact" OR “tissue use” )  AND  intervention
